# Age-related differences in immune responses to inactivated influenza and adjuvanted recombinant herpes zoster vaccines

**DOI:** 10.1172/jci.insight.195618

**Published:** 2026-06-02

**Authors:** Gizem Kilic, Esther J.M. Taks, Leonie S. Helder, Elisabeth A. Dulfer, Büsra Geckin, Liesbeth van Emst, Heidi Lemmers, Stefano Berrè, Adhidev Biswas, Mumin Ozturk, Yutaka Negishi, Wivine Burny, Sofia M. Buonocore, Jaap ten Oever, Musa M. Mhlanga, Mihai G. Netea

**Affiliations:** 1 Department of Internal Medicine and Radboud Community for Infectious Diseases, Radboud University Medical Center, Nijmegen, Netherlands.; 2 GSK, Rixensart, Belgium.; 3 GSK, Bengaluru, India.; 4 Department of Cell Biology, Faculty of Science, Radboud University, Nijmegen, Netherlands.; 5 Department of Human Genetics, Radboud University Medical Center, Nijmegen, Netherlands.; 6 Department of Immunology and Metabolism, Life and Medical Sciences Institute, University of Bonn, Bonn, Germany.

**Keywords:** Aging, Immunology, Infectious disease, Influenza, Vaccines

## Abstract

Immunosenescence, the biological aging of the immune system, leads to dysregulated immune responses, increasing susceptibility to infections and reducing vaccine efficacy in older adults, as seen with flu vaccines. In contrast, the AS01-adjuvanted recombinant herpes zoster vaccine (RZV) maintains high and sustained efficacy, offering 82% protection against herpes zoster at 11 years after vaccination in individuals over 50. To identify factors affecting age-dependent vaccine efficacy, we conducted a randomized, partially placebo-controlled clinical study. Young adults (18–35 years, *n* = 84) were randomized 3:3:1:1 to receive either RZV, an inactivated quadrivalent seasonal influenza vaccine (IIV4), or a placebo for RZV or for IIV4, and older adults (≥60, *n* = 63) were randomized 1:1 to receive RZV or IIV4. RZV elicited robust antibody production, antigen-specific polyfunctional CD4^+^ T cell responses, and IFN-γ from PBMCs in both age groups, while IIV4 increased antibody responses but induced fewer antigen-specific CD4^+^ T cells and no elevation of IFN-γ from PBMCs. Interestingly, RZV reduced systemic inflammation in older adults, particularly after the second injection. Baseline inflammation negatively correlated with antibody production and IFN-γ response, especially after RZV. Our findings suggest that RZV may help overcome immunosenescence by enhancing cellular responses and potentially decreasing systemic inflammation, deserving further investigation into the underlying molecular mechanisms.

## Abstract 2

The recombinant zoster vaccine worked well in older adults, boosting immune responses and lowering inflammation, even with aging of the immune system.

## Introduction

Vaccination remains the most powerful and effective strategy for protection against potentially severe infections. Over the years, various vaccine technologies have been developed, including live-attenuated, subunit, inactivated, and mRNA-based vaccines. Despite their distinct development methods, all vaccines share a common goal: to administer a less harmful dose or form of antigens to the host, enabling the immune system to recognize and build a robust, specific response to future exposure with the respective pathogen. Vaccines are estimated to have saved 154 million lives between 1974 and 2024 (1) and continue to protect countless individuals from severe diseases. However, vaccine effectiveness greatly varies across individuals and populations.

Vaccine effectiveness is influenced by various factors, including host factors such as age, sex, and genetic background; perinatal factors such as maternal antibodies; and external factors, such as seasonal variations and past infections (2). Among these factors, aging is particularly notable for its impact on vaccine-induced immune responses. An aging innate immune system displays a bias toward myelopoiesis, yet shows impaired functional innate immune responses including lower capacity for phagocytosis, cytotoxicity, and ROS production (3). In adaptive immunity, aging results in a decline of T and B cell responses and a reduced pool of naive lymphocytes, which are essential for recognizing new antigens (4, 5). This reduction limits the ability of the immune system to respond to infections and diminishes the effectiveness of vaccines. Additionally, memory T and B cells, which are essential for long-term immunity, experience functional decline, further compromising the immune response. Finally, circulating inflammatory protein concentrations increase with age, contributing to low-grade systemic inflammation and an increased susceptibility to age-related inflammatory disorders (6).

All these changes result in higher susceptibility to infections and lower vaccine efficacy in older individuals. For instance, the efficacy of many vaccines, like those for SARS-CoV-2 and influenza, is generally lower in older adults than in young adults (7, 8). According to a recent test-negative design study, in which vaccination status was compared between patients who tested positive versus negative for influenza, the effectiveness of the annual influenza vaccines against any influenza was 49% for children (<18 years), 37% for young adults (18–64 years), and 31% for older adults (≥65 years) (9). The most commonly used seasonal influenza vaccines consist of 3 or 4 inactivated viral strains, are non-adjuvanted, and are a single dose. Notably, the effectiveness of the seasonal influenza vaccine varies each year, partly depending on how closely the circulating strains match the strains included in the vaccine (10). Moreover, the segmented, negative-sense RNA genome of the virus allows genetic reassortment, which leads to antigenic drift and shift (11). These processes drive frequent and unpredictable changes in viral surface antigens, contributing to reduced effectiveness of the vaccine within a given season.

In contrast, an adjuvanted recombinant herpes zoster vaccine (RZV), licensed for use in 2017, has shown high efficacy in older adults. RZV, combining the recombinant glycoprotein E (gE) of the varicella zoster virus (VZV) with the adjuvant AS01, has demonstrated 97.2% efficacy during a mean follow-up period of 3.2 years, 90.9% efficacy at 7 years, and 82% at 11 years in preventing herpes zoster in adults 50 years and older (12–14). In people over 70 years of age, vaccine efficacy was 91.3% at 3.2 years after immunization (15). The vaccine is administered in 2 doses, separated by 2–6 months, and it is well tolerated, with an acceptable safety profile for both young and older adults (16, 17). It is unclear how RZV is able to achieve such high efficacy despite an immunosenescent state, but it is hypothesized that the adjuvant itself and the synergy between the adjuvant and antigen stimulate a persistent response, even in immunocompromised or frail individuals (18). The molecular substrate of this effect is not yet known.

To understand the molecular mechanisms and immunological pathways contributing to age-dependent vaccine effectiveness, we conducted a randomized and partially placebo-controlled vaccination trial in young (18–35 years) and older (≥60 years) adults who received either IIV4, RZV, or a placebo. IIV4 is widely used in older populations but shows variable, age-dependent efficacy, making it suitable for studying reduced protection with age. In contrast, RZV remains highly effective in older adults, and exploring the underlying mechanisms leading to high protection will give valuable information into how it overcomes age-related decline in immune function.

In this substudy of the clinical trial, we evaluated the immunological differences between age groups by measuring immune cell subsets; neutralizing antibody titers; PBMC-derived IFN-γ production and CD4^+^ T cell responses to the specific vaccine antigens; and concentrations of circulating inflammatory proteins before and after vaccination. Finally, we explored how baseline systemic inflammation is associated with vaccine-induced immune responses.

## Results

### Safety data and participant demographics.

**Figure 1:**
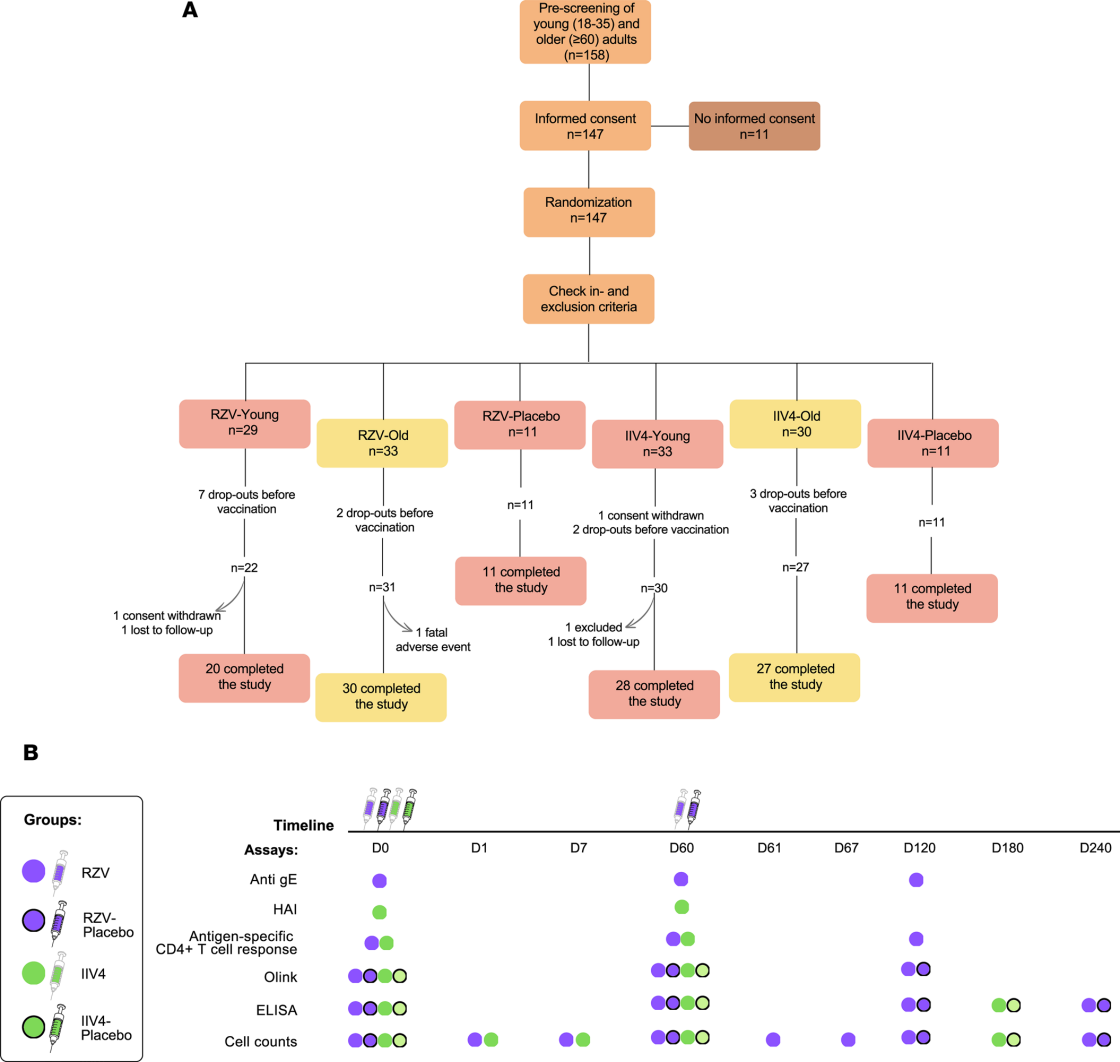
Summary of the clinical study and the time points of each assay. (**A**) The flow diagram depicts participant recruitment, randomization, and the number of people who started and finished the study. (**B**) Vaccination groups in the study and the time points for each injection and experiment.

For the study, 84 young and 63 older adults were recruited. After the initial screening and informed consent process, participants were randomized into different study arms based on age and vaccine type, as illustrated in Figure 1A. Because of the extended period between the screening/randomization and vaccination visits (D0), some participants dropped out or withdrew their consent. Ultimately, 22 young and 31 older participants were immunized with RZV, and 30 young and 27 older adults received IIV4. Additionally, 11 young adults in the placebo-IIV4 group and 11 adults in the placebo-RZV group were vaccinated with a placebo. The timeline, sample collection, and performed assays are summarized in Figure 1B. The demographics, comorbidities, and risk factors of the study cohort are presented in Table 1. As expected, older adults had more comorbidities and risk factors, such as hypertension and chronic cardiovascular disease.

Overall, both vaccines were well tolerated, showing acceptable safety profiles in young and older participants. The most common local adverse reaction within the first week after vaccination was pain at the injection site, and the most common systemic adverse reactions were myalgia and fatigue (Supplemental Tables 1–3; supplemental material available online with this article; https://doi.org/10.1172/jci.insight.195618DS1). Most of the adverse reactions were rated mild or moderate by the study participants, whereas some severe (grade 3) adverse reactions were also reported (Supplemental Tables 4–6). These adverse reactions typically resolved spontaneously within a week. Three serious adverse events (SAEs) were recorded during the study, which were a road traffic accident (RZV-young group), atrial fibrillation (RZV-old), and acute heart failure (RZV-old), the last SAE being fatal (Supplemental Table 7). Given that the participants with atrial fibrillation and acute heart failure both had a prior history of similar conditions and were receiving related medications, and the participant involved in the traffic accident did not develop any condition that contributed to the accident, the SAEs were not considered related to the study vaccines.

### Baseline differences in immune parameters between young and older adults.

We first evaluated the immune system parameters in young and older adults before they received any study vaccination. The numbers of WBCs, neutrophils, monocytes, and lymphocytes were similar between the 2 age groups (Supplemental Figure 1A). Additionally, the baseline concentrations of anti-VZV gE antibodies and the HA inhibition (HAI) titers against the A/Victoria/H1N1 and B/Phuket virus strains were also comparable in young and older adults (Supplemental Figure 1, B and C). In contrast, the IFN-γ production of PBMCs from young adults was significantly higher after a 7-day incubation with the IIV4 vaccines and gE antigen compared with that from older counterparts (Supplemental Figure 1D). Lastly, many inflammatory proteins were significantly more abundant in the circulation of older individuals than in young volunteers (Supplemental Figure 1E).

### IIV4-induced modulation of immune cell numbers, HAI titers, antigen-specific CD4^+^ T cells, and IFN-γ production in young and older adults.

**Figure 2:**
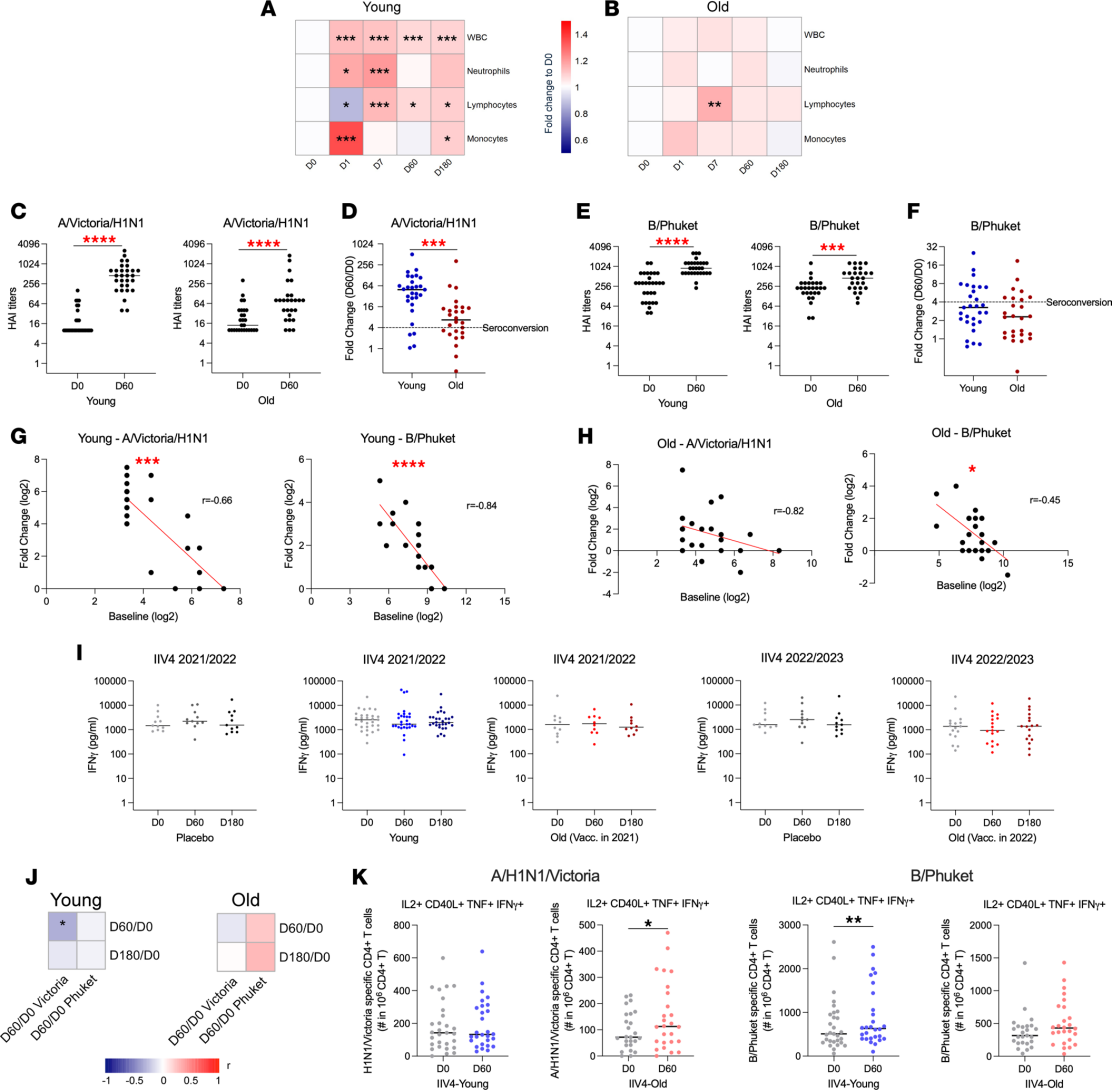
IIV4-induced changes in immune cell counts and adaptive immune responses in young and older adults. Heatmaps showing the fold changes in immune cell counts after IIV4 vaccination versus before vaccination in the (**A**) young and (**B**) older groups. Fold changes at D1, D7, D60, and D180 were compared with D0, after correction for multiple testing using the Benjamini-Hochberg method. The scale displays fold change values; stars on the heatmap represent FDR values. (**C**) HAI titers and (**D**) comparison of fold changes in antibody production against the A/Victoria/H1N1 strain. (**E**) HAI titers and (**F**) comparison of fold changes (D60/D0) in antibody production against the B/Phuket strain. Spearman’s correlation between baseline HAI titers against the A/Victoria/H1N1 and B/Phuket strains and fold changes after IIV4 vaccination in (**G**) young and (**H**) older adults. (**I**) IFN-γ production from PBMCs after 7-day stimulation with 1 μg/mL of IIV4 2021/2022 and 2022/2023 season vaccines. PBMCs from each individual were stimulated with the same season of the vaccine they received. (**J**) Spearman’s correlation of fold IFN-γ response (D60/D0) and fold HAI titers (D60/D0) for the A/Victoria/H1N1 and B/Phuket strains. The scale indicates the correlation coefficient (*r*). (**K**) The frequency of activated (4-1BB+) CD4^+^ T cells per 10^6^ CD4^+^ T cells that were positive for CD40L, IL-2, TNF, and IFN-γ after stimulation with A/H1N1/Victoria and B/Phuket. The *y* axis values in **C**–**F** are displayed on a log_2_ scale; values in **I** are shown on a log_10_ scale. The dashed lines on graphs in **D** and **F** indicate the threshold of seroconversion (a fold change of 4). Different time points within the same group were compared using Wilcoxon’s signed-rank test; fold changes between young and older adults were compared using the Mann-Whitney *U* test. *P < 0.05, **P < 0.01, ***P < 0.001, ****P < 0.0001.

Next, we analyzed changes in circulating immune cells after IIV4 vaccination compared with baseline (D0). In young adults, immune cell counts, except lymphocytes, increased 1 day after vaccination (Figure 2A). Although lymphocyte numbers decreased on D1, they remained significantly elevated for the remainder of the study compared with the baseline. WBC counts remained higher on day 180 compared with the baseline, possibly due to the higher numbers of lymphocytes and monocytes. In older adults, changes were minimal (Figure 2B). Absolute cell counts after IIV4 and placebo vaccination are presented in Supplemental Figure 2, A–C.

Subsequently, we measured HAI titers against the viral strains in the 2021/2022 and 2022/2023 IIV4 vaccines before and 60 days after vaccination. Both age groups showed a significant increase in anti-A/Victoria/H1N1 titers, with a stronger rise in young adults (Figure 2, C and D). Similarly, IIV4 also boosted anti-B/Phuket titers in both groups, and the fold induction was not significantly different between the groups (Figure 2, E and F). Notably, the fold HAI induction to the A/Victoria/H1N1 and B/Phuket strains was significantly higher in older adults vaccinated with the IIV4 2021/2022 compared with the 2022/2023 season vaccine (Supplemental Figure 2D).

Seroprotection refers to an antibody titer of 1:40 or higher, a level associated with a 50% reduction in influenza risk (19). In our study, 23% of young adults had HAI titers of 40 or higher to A/Victoria/H1N1 before vaccination, which increased to 100% on D60. In older adults, the corresponding rates were 30% before vaccination and 73% after vaccination. All young adults had titers of 40 or higher against B/Phuket both before and after vaccination. Among older adults, 93% had titers of 40 or higher, rising to 100% after receiving IIV4.

For the A/Tasmania/H3N2 and B/Washington strains (present only in the IIV4 2021/2022 vaccine), the HAI titers against A/Tasmania/H3N2 increased only in the young group, but seroconversion rates were extremely low in the 2 age groups (Supplemental Figure 2, E and F). Both young and older adults showed significant antibody increases against B/Washington with similar fold changes (Supplemental Figure 2, G and H). No notable changes were observed in antibody levels against the A/Darwin/H3N2 and B/Austria strains 60 days after vaccination (Supplemental Figure 2I).

Previous studies have shown that preexisting immunity, defined by the presence of specific antibodies before vaccination, negatively correlates with a fold antibody induction after vaccinations like those for influenza, herpes zoster, and pneumococcus (20–22). As expected, we observed a negative correlation between prevaccination HAI titers and antibody response for A/Victoria/H1N1 and B/Phuket strains in young adults (Figure 2G). In the older group, a moderate-to-high negative correlation was also observed, though not significant for A/Victoria/H1N1 (Figure 2H). As such, age may independently affect the relationship between prevaccine and postvaccine responses. Additionally, IFN-γ production in PBMCs after 7 days of incubation with IIV4 was similar before and after vaccination across all groups (Figure 2I). Subsequently, we explored the potential link between PBMC-derived IFN-γ production and antibody response to IIV4. Fold induction of A/Victoria/H1N1 HAI titers and IFN-γ responses were negatively correlated at D60 in younger adults (Figure 2J).

Last, we determined the frequency of antigen-specific activated CD4^+^ T cells that express CD40L, IL-2, TNF, and IFN-γ after IIV4 in young and older adults (Supplemental Figure 3, A and B). In general, the induction of activated antigen-specific CD4^+^ T cells by IIV4 was lower in older adults compared with the young individuals. CD40L was the most expressed marker upon incubation with the A/H1N1/Victoria strain. IIV4 led to a small but significant induction of B/Phuket-specific CD4^+^ T cells that were positive for 2 or 3 markers. There was a significant increase in CD4^+^ T cells expressing all 4 markers (CD40L, IL-2, TNF, and IFN-γ) in response to A/H1N1/Victoria only in older adults, whereas such an increase in response to B/Phuket was observed only in young adults (Figure 2K).

Overall, young adults showed higher antibody responses to IIV4, but the vaccine did not enhance IFN-γ production from PBMCs in either age group. The vaccine induced polyfunctional antigen-specific CD4^+^ T cells, although the cell frequencies were low, suggesting the limited ability of IIV4 to boost cellular responses.

### RZV-induced modulation of immune cell numbers, antibody concentrations, antigen-specific CD4^+^ T cells, and IFN-γ production in young and older adults.

**Figure 3:**
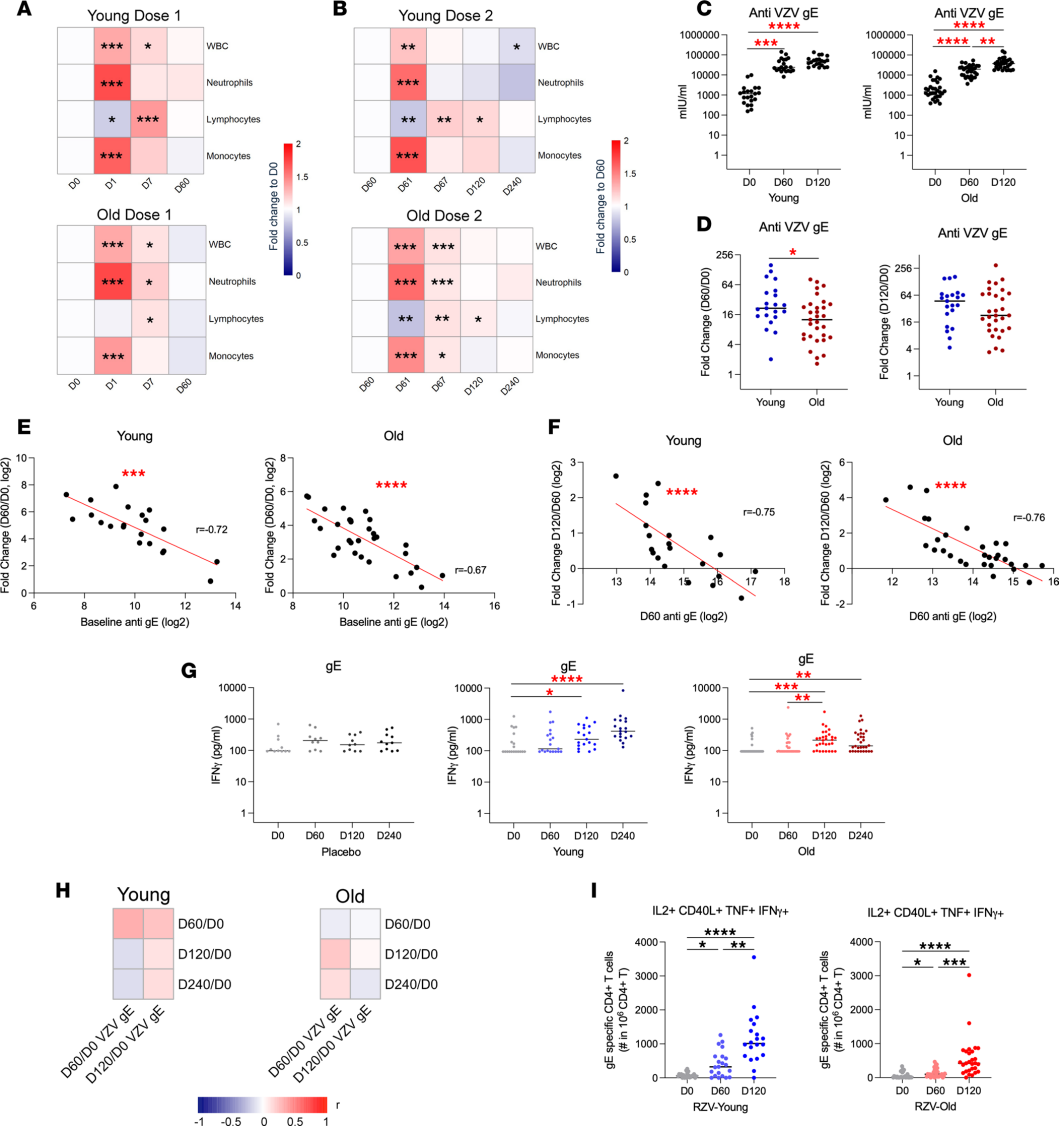
RZV-induced changes in immune cell counts and adaptive immune responses in young and older adults. Heatmaps showing the fold changes in immune cell counts after the (**A**) first and (**B**) second dose of RZV. Fold changes at D1, D7, and D60 were compared with D0; fold changes at D61, D67, D120, and D240 were compared with D60 using Wilcoxon’s signed-rank test, with *P* values corrected for multiple testing using the Benjamini-Hochberg method. The scale displays fold change values; stars on the heatmap represent FDR values. (**C**) Anti-HZV gE antibody concentrations and (**D**) comparison of fold changes (D60/D0 and D120/D0) after RZV in young and older individuals. (**E**) Spearman’s correlation between baseline concentrations of anti-VZV gE antibodies and fold antibody response (D60/D0) after RZV. (**F**) Spearman’s correlation between pre-second vaccination (D60) anti-VZV gE antibody concentrations and fold antibody response (D120/D60) after second dose. (**G**) IFN-γ production from PBMCs after 7-day stimulation with 4 μg/mL of the antigen (gE) in RZV. (**H**) Spearman’s correlation of fold IFN-γ responses (D60/D0 and D120/D0 and D240/D0) and fold antibody production (D60/D0 and D120/D0). The scale indicates the correlation coefficient (*r*). (**I**) The frequency of activated (4-1BB+) CD4^+^ T cells per 10^6^ CD4^+^ T cells that were positive for CD40L, IL-2, TNF, and IFN-γ after stimulation with the gE antigen in young and older adults. The *y* axis values in **C** and **G** are displayed on a log_10_ scale; values in **D** are shown on a log_2_ scale. Different time points within the same group were compared using Dunn’s multiple-comparison test, and fold changes between young and older adults were compared using the Mann-Whitney *U* test. *P < 0.05, **P < 0.01, ***P < 0.001, ****P < 0.0001.

We characterized how RZV affected immune cell counts after the first and second doses. RZV induced greater fold changes in cell numbers than IIV4. After the first dose, both young and old groups experienced temporary increases in WBCs, neutrophils, and monocytes on day 1, with over a 1.7-fold increase in neutrophils (Figure 3A). Lymphocyte numbers decreased on D1 only in the young group. Similar changes occurred after the second dose, with significant lymphocyte decreases on day 1 (D61) in both groups, followed by a rise on D67 that stayed elevated until at least D120 (Figure 3B). Absolute numbers of cells after RZV and placebo vaccinations are presented in Supplemental Figure 4, A–C.

RZV led to strong antibody production after the first dose in both age groups (Figure 3C). The second dose further increased anti-VZV gE antibody levels, though the increase in young adults did not reach statistical significance. Young adults showed a greater fold antibody increase only after the first dose (Figure 3D). We also found that prevaccination antibody concentrations were negatively correlated with a fold increase in antibodies after RZV in both age groups (Figure 3E). The concentrations of antibodies before the second vaccination (D60) were negatively associated with fold antibody increase after the second vaccination (Figure 3F).

IFN-γ production after gE antigen stimulation increased significantly after 2 RZV doses (D120) and remained elevated at D240, unlike the placebo, which had no effect (Figure 3G). Importantly, prior to RZV, IFN-γ response was detectable in 55% of the young participants, increasing to 92% after the second dose (D120). In older adults, it increased from 20% at baseline to 77% on D120. No significant relationship was found between IFN-γ and antibody production after RZV exposure (Figure 3H).

RZV induced a robust and significant increase in antigen-specific polyfunctional CD4^+^ T cells in both age groups, with a lesser degree in the older age group (Supplemental Figure 5). The second dose further increased the frequency of antigen-specific cells. CD40L was the most expressed marker to the antigen among the 4 markers measured. CD40L^+^IL-2^+^TNF^+^IFN-γ^+^ gE–specific CD4^+^ T cells had the highest frequency among other marker combinations and were significantly induced after both the first and second RZV dose in young and older adults (Figure 3I). In summary, RZV elicited significant humoral and cellular responses in both young and older individuals.

### The impact of IIV4 on circulating immune mediators.

**Figure 4:**
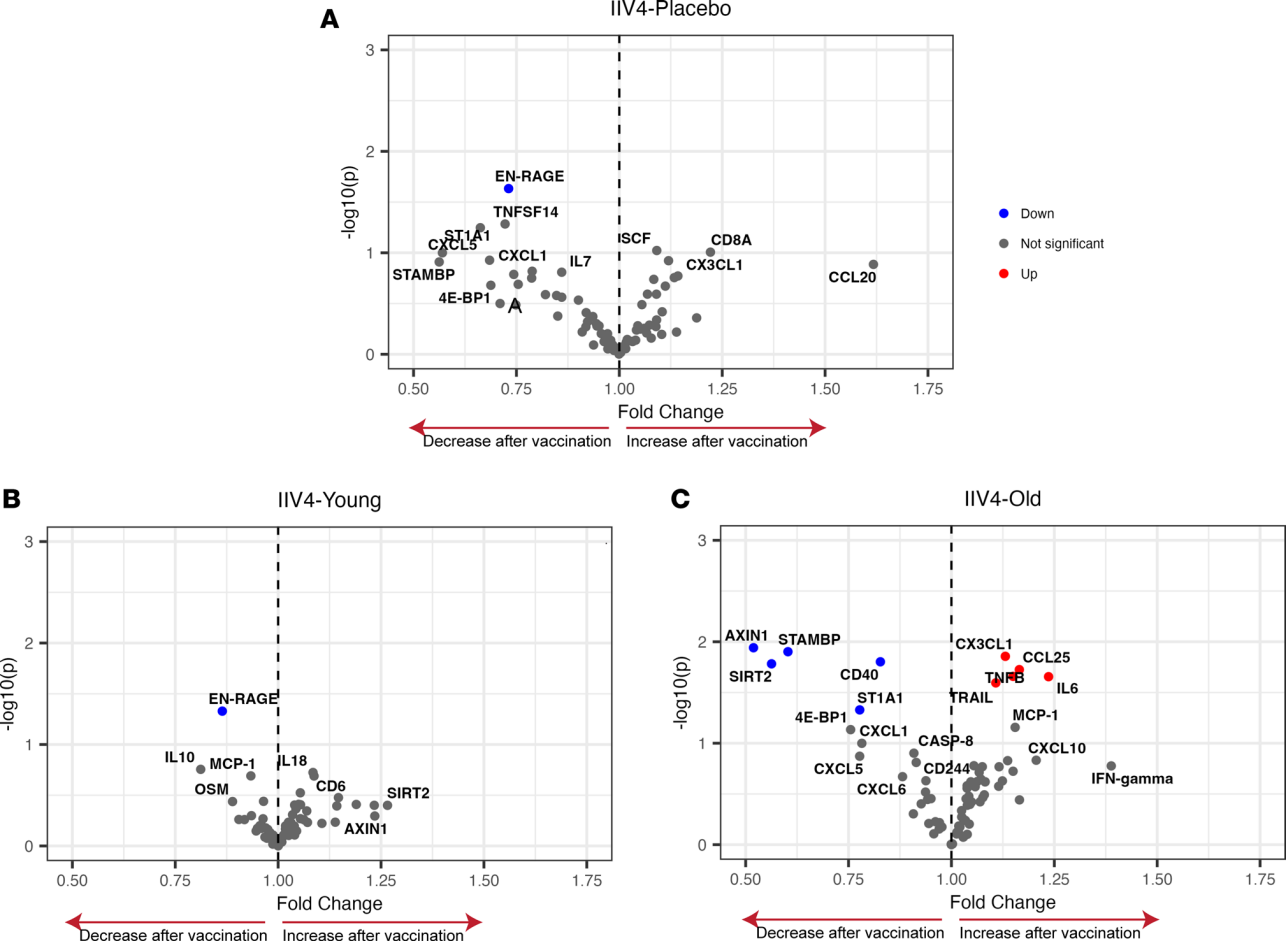
IIV4-induced changes in circulating protein concentrations in young and older adults. Volcano plots displaying the changes in proteins in the (**A**) placebo group, (**B**) young adults, and (**C**) older adults 60 days after vaccination. Blue dots represent the significantly downregulated proteins after vaccination; red dots show the significantly upregulated ones. A moderated 2-tailed *t* test was used in the R package *limma* to compare protein concentrations before and after vaccination (nominal *P* < 0.05).

We analyzed the impact of IIV4 and a placebo on circulating inflammatory proteins using the Olink 96 Target Inflammation Panel. At 60 days after vaccination, EN-RAGE levels decreased in both the IIV4 placebo and young groups (Figure 4, A and B). In older adults, IIV4 vaccination increased inflammatory mediators such as TNFB, IL-6, CCL25, and TRAIL, while reducing AXIN1, STAMBP, and CD40 (Figure 4C and Supplemental Figure 6A). Since older adults received either the 2021/2022 or the 2022/2023 season of the IIV4 vaccine, we analyzed the effect of these 2 vaccines separately. Interestingly, the 2021/2022 vaccine increased oncostatin M (OSM) and decreased EN-RAGE (Supplemental Figure 6B), while the 2022/2023 vaccine affected other proteins without affecting OSM or EN-RAGE (Supplemental Figure 6, C and D). These results suggest IIV4 has minimal impact on inflammatory proteins in young adults but varies in older adults depending on the vaccine year.

### The association between baseline inflammation and adaptive immunity responses to IIV4.

**Figure 5:**
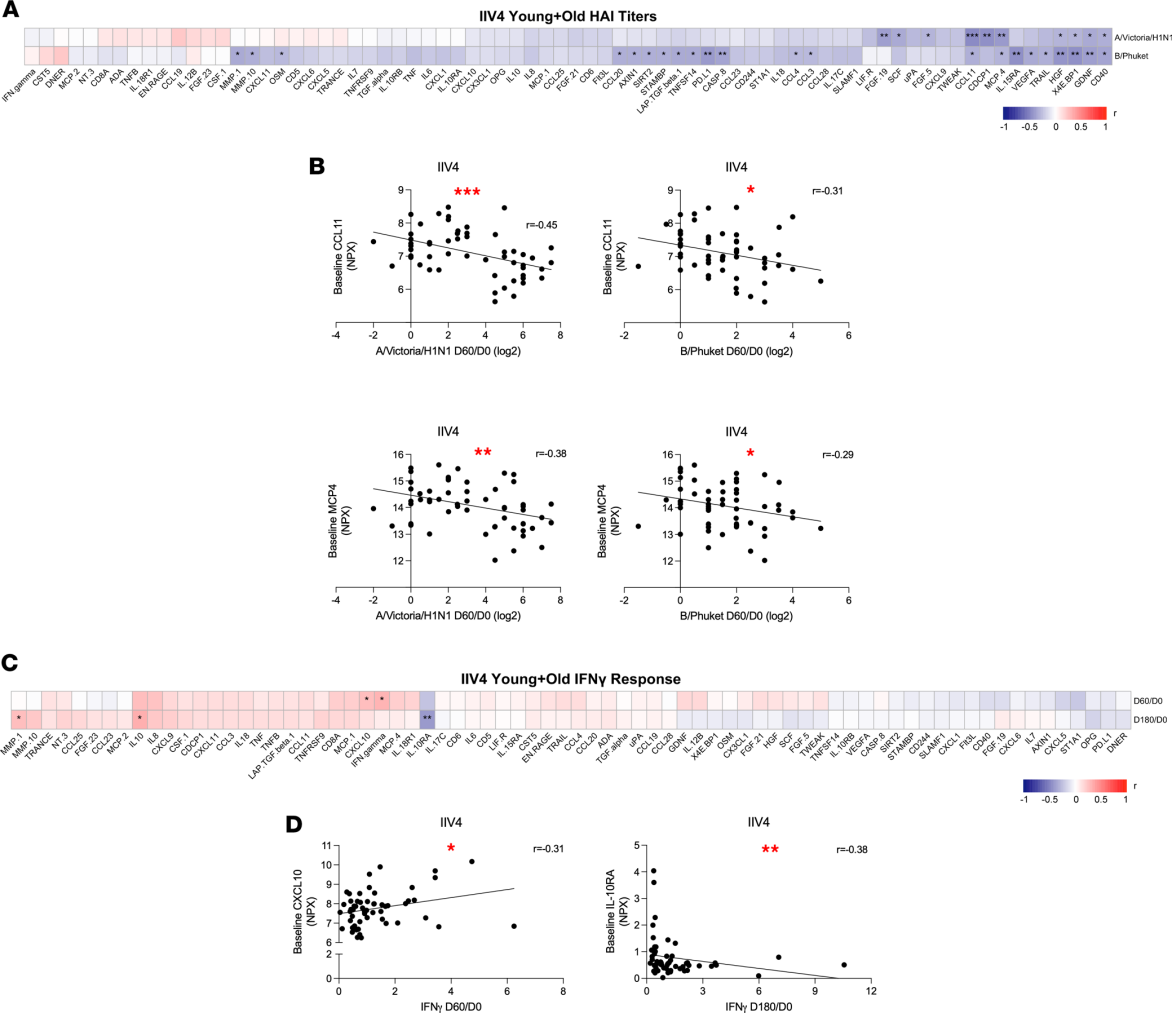
Correlation between circulating protein concentrations at baseline and IIV4-induced immune responses across combined age groups. (**A**) Heatmap depicting the association between baseline concentrations of circulating immune-related proteins and the fold antibody response (D60/D0) against the A/Victoria/H1N1 and B/Phuket strains after IIV4 vaccination. (**B**) Example correlation plots showing the relationship between CCL11 and MCP4 with the fold HAI titers to A/Victoria/H1N1 and B/Phuket. (**C**) Heatmap illustrating the correlation between baseline circulating immune-related proteins and the fold IFN-γ response (D60/D0 and D180/D0) from PBMCs after 7-day stimulation with 1 μg/mL of IIV4. (**D**) Example correlation plots of baseline CXCL10 levels with D60/D0 fold IFN-γ induction, and IL-10RA with D180/D0 fold IFN-γ production. Spearman’s correlation was used; the scales in **A** and **C**, along with the *r* values on the correlation graphs, represent the Spearman’s correlation coefficients. Stars on heatmaps indicate the nominal *P* values. NPX, normalized protein expression. *P < 0.05, **P < 0.01, ***P < 0.001.

The immune system’s baseline status before vaccination is crucial to predicting vaccine responses (23). Thus, we examined the relationship between baseline inflammation levels as assessed by circulating proteins and the antibody/IFN-γ responses to vaccination in both age groups combined and separately. In the IIV4 cohort, higher baseline inflammation was linked to lower fold-increases in A/Victoria/H1N1 and B/Phuket HAI titers, with proteins like CCL11, MCP-4, and HGF negatively associated with antibody responses (Figure 5, A and B).

Age-specific analysis showed baseline inflammation negatively linked to B/Phuket HAI responses in both age groups in general, while A/Victoria/H1N1 showed mixed associations (Supplemental Figure 7, A–C). No significant overlapping proteins were found in young and older adults.

Unlike neutralizing antibody titers, fold changes in IFN-γ were positively correlated with baseline systemic inflammation when both groups were combined (Figure 5, C and D). Higher baseline concentrations of IL-10RA were, however, linked to lower IFN-γ increases in the young group (Supplemental Figure 7D). In older adults, baseline inflammatory proteins like MCP-4, IL-8, CXCL9, and CXCL10 were positively associated with IFN-γ responses (Supplemental Figure 7, E and F).

Our results indicate that baseline inflammation is generally negatively associated with antibody production and positively linked with specific cellular IFN-γ responses to viral strains in IIV4. However, no distinct patterns were observed between age groups.

### The impact of RZV on circulating immune mediators.

**Figure 6:**
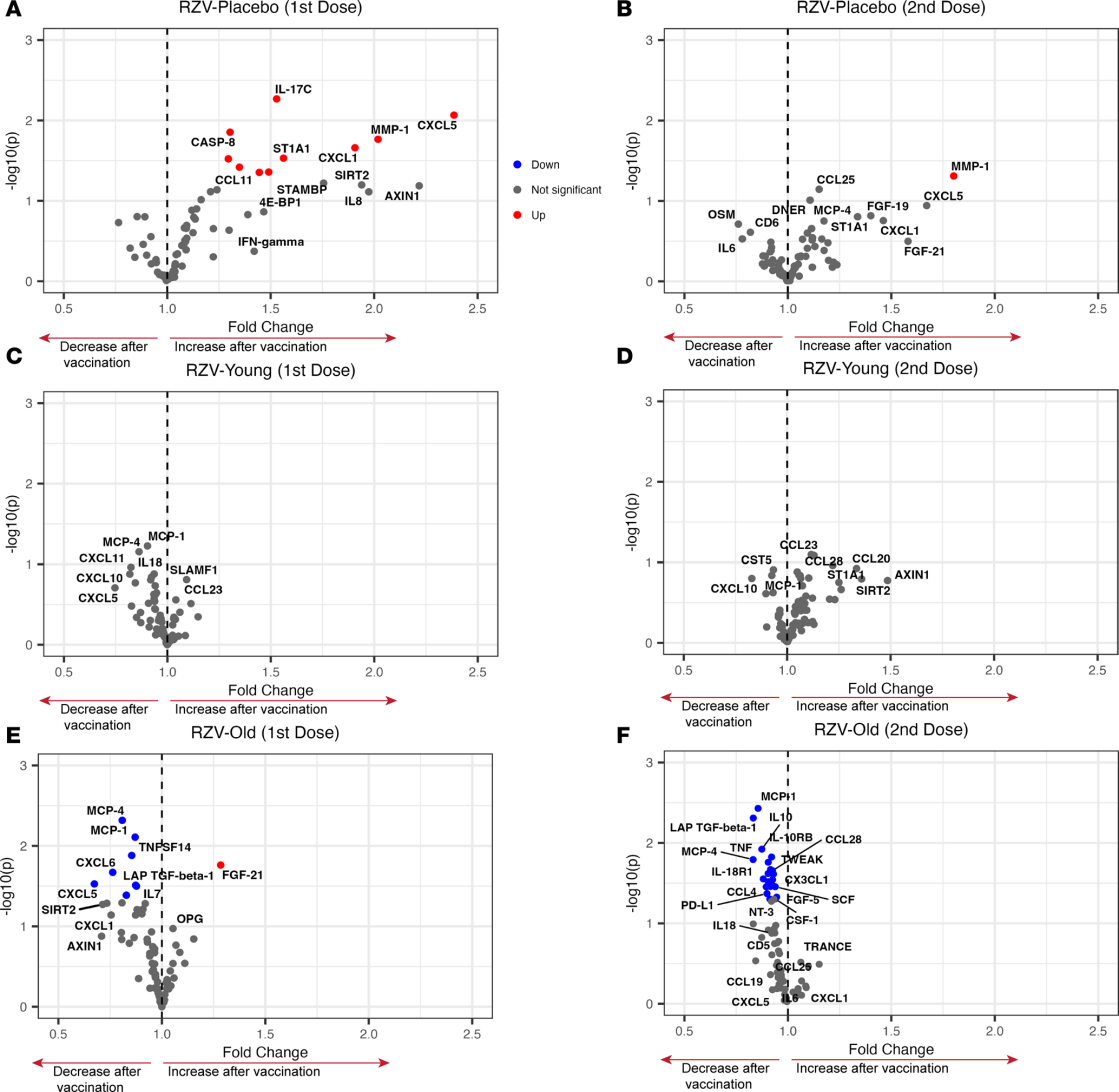
RZV-induced changes in circulating protein concentrations in young and older adults. Volcano plots displaying the changes in proteins in the (**A** and **B**) placebo group, (**C** and **D**) young adults, and (**E** and **F**) older adults after the first or second dose of vaccination compared with baseline (D0). **A**, **C**, and **E** exhibit the protein fold change after the first dose (D60/D0); **B**, **D**, and **F** show the protein fold change after the second dose (D120/D0). Blue dots represent the significantly downregulated proteins after vaccination; red dots show the significantly upregulated ones. A moderated 2-tailed *t* test was used in the R package *limma* to compare protein concentrations before and after vaccination (nominal *P* < 0.05).

Next, we analyzed the changes in circulating proteins after the placebo and RZV injections. After the first placebo injection, proteins like IL-17C, CXCL5, CXCL1, MCP-4, and MMP-1 increased, but only MMP-1 remained elevated after the second dose (Figure 6, A and B, and Supplemental Figure 8A). RZV had no notable influence on the investigated proteins in young adults (Figure 6, C and D). Interestingly, in older individuals, the first RZV dose reduced proteins such as MCP-1, MCP-4, CXCL5, and CXCL6, while the second dose broadly downregulated inflammation-related proteins like IL-10, TNF, IL-18R1, and TWEAK, among others (Figure 6, E and F, and Supplemental Figure 8B). These results suggest RZV reduces systemic inflammation in older adults.

### The association between baseline inflammation and adaptive immune responses to RZV.

**Figure 7:**
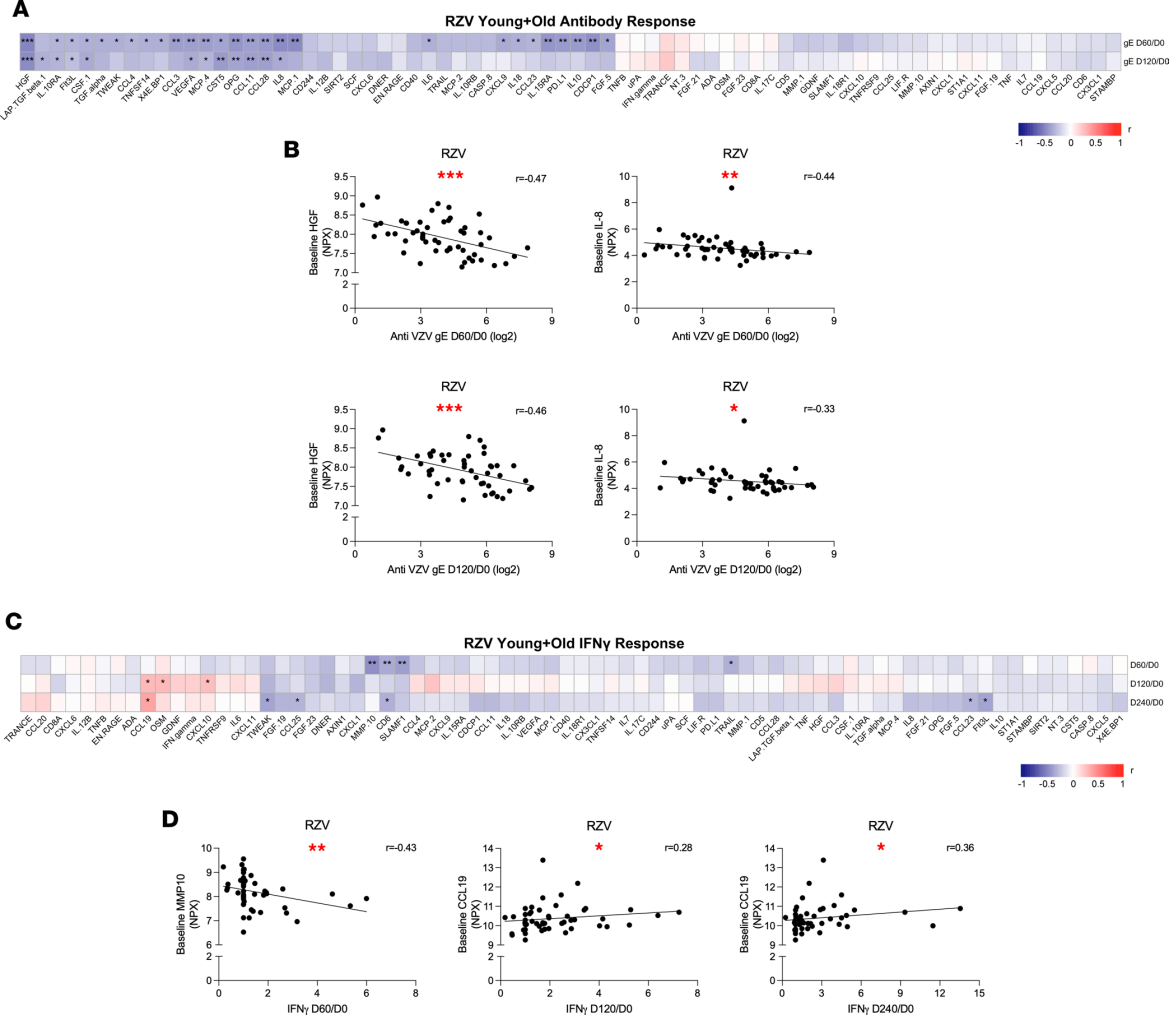
Correlation between circulating protein concentrations at baseline and RZV-induced immune responses across age groups. (**A**) Heatmap depicting the association between baseline concentrations of circulating immune-related proteins and the fold antibody response (D60/D0 and D120/D0) after RZV. (**B**) Example correlation plots showing the relationship between baseline levels of HGF and IL-8 with the fold antibody response after the first (D60/D0) and second dose (D120/D0). (**C**) Heatmap illustrating the correlation between baseline circulating immune-related proteins and the fold IFN-γ response (D60/D0, D120/D0, and D240/D0) from PBMCs after 7-day stimulation with 4 μg/mL of the gE antigen in RZV. (**D**) Example correlation plots of baseline MMP10 levels with D60/D0 fold IFN-γ, and CCL19 with D120/D0 and D240/D0 fold IFN-γ production. Spearman’s correlation was used; the scale in **A** and **C**, along with the *r* values on the correlation graphs, represent Spearman’s correlation coefficients. Stars on heatmaps indicate the nominal *P* values. NPX, normalized protein expression. *P < 0.05, **P < 0.01, ***P < 0.001.

Last, we evaluated the relationship between baseline inflammation and immune responses to RZV. Overall, higher levels of circulating immunomodulatory proteins were negatively correlated with antibody responses, even after 2 doses (Figure 7, A and B). Proteins like HGF, IL-8, IL-10RA, CSF-1, and OPG were associated with lower antibody responses in both age groups.

Age-specific analysis showed stronger negative correlations in young adults, with proteins like CCL4 and CCL11 linked to lower antibody responses. In older adults, correlations were mixed; higher levels of CXCL9, CXCL10, and IFN-γ were associated with greater antibody induction (Supplemental Figure 9, A–C).

Baseline concentrations of circulating immune mediators were generally negatively correlated with IFN-γ responses at D60 and D240 after RZV, although some positive associations emerged after the second dose (D120/D0) (Figure 7C). Notably, higher baseline concentrations of CD6 and MMP10 were moderately linked to reduced IFN-γ response to the gE antigen after the first dose. In contrast, elevated CCL19 correlated with an increased IFN-γ fold change at D120 and D240 (Figure 7D).

When the age groups were separated, different patterns were observed (Supplemental Figure 9, D and E). In the RZV-young group, higher TWEAK concentrations consistently correlated with weaker IFN-γ responses (Supplemental Figure 9E). Mirroring the overall trend observed in the age-combined RZV group, baseline CD6 was negatively linked to IFN-γ response at D60 in the old group, while CCL19 was positively linked to IFN-γ at D120 and D240 (Supplemental Figure 9F).

Overall, baseline inflammation was negatively associated with RZV-induced adaptive responses when age groups were combined. However, when analyzed separately, different correlation patterns were seen, with older adults displaying a mix of responses, including more positive associations.

## Discussion

Aging substantially affects vaccine responses, with many vaccines being less effective in older adults. Adjuvants have been developed to address this by enhancing immune activation and memory (24). In this study, we evaluated age-dependent differences in immune system responses to IIV4 and RZV. The results are summarized in Table 2. RZV induced strong antibody and IFN-γ responses in both age groups and reduced systemic inflammation in older adults. In contrast, IIV4 primarily increased neutralizing titers, stronger in young adults, but failed to induce effective cellular responses.

These results align with previous reports of strong cellular and humoral responses induced by RZV in both age groups and weak or no cellular responses from seasonal influenza vaccines (17, 25, 26). Complementing previous findings, we found that both vaccines were well tolerated, with an acceptable safety profile in both age groups (13, 27). Although this is a small cohort, it represents valuable information, as safety data on RZV in young adults has been limited compared with older adults (28).

The efficacy of vaccines is primarily mediated through the induction of specific antibodies and T cell responses. T cell responses play a key role in fighting infections and providing long-term protection. For example, memory T cells are especially important in controlling varicella zoster virus infections, which cause shingles (29). In addition, antigen-specific CD4^+^ T cells and IFN-γ production from these cells have been linked to protection against influenza, but their numbers decrease significantly in older adults (30). IIV4’s inability to enhance cellular responses likely contributes to its suboptimal effectiveness. An important function of adjuvants is to enhance the T cell memory response (31). For instance, AS01 in RZV contains 2 components: the TLR4 ligand monophosphoryl lipid A (MPLA) and saponin QS-21 derived from the *Quillaja* *saponaria* tree (32). MPLA induces the production of proinflammatory cytokines through the MyD88 pathway, and QS-21 activates the inflammasome and IL-18/IL-1β release. The synergistic effect of these 2 components is essential to achieve an optimal T cell response (33). Overall, AS01 triggers strong innate activation in draining lymph nodes via monocytes/macrophages, DCs, and NK cells and early release of IFN-γ in an IL-12/IL-18–dependent manner (33, 34).

Clinical studies with AS01, such as those done with tuberculosis and malaria vaccines, demonstrated strong antigen-specific CD4^+^ T cell responses and antibody production (35, 36). In this study, we found an RZV-induced robust increase in polyfunctional antigen-specific CD4^+^ T cells that express different combinations of CD40L, IL-2, TNF, and IFN-γ in both age groups. Notably, T cell polyfunctionality is linked to protection by various vaccines, such as those for COVID-19 and yellow fever (37, 38). Additionally, CD4^+^ T cell–mediated immunity after RZV was associated with better antibody responses for at least 3 years after vaccination (39). Because AS01 induces a robust and durable T cell response, its use could be further explored in vaccines targeting pathogens where strong cellular immunity is essential, as well as in populations with weakened immunity, including immunocompromised or older individuals. Interestingly, the type and magnitude of immune responses induced by AS01-adjuvanted vaccines vary across different antigens, suggesting that both the adjuvant and antigen contribute to forming the overall immunological profile (32).

An important finding of our study is that both vaccines induce CD40L on antigen-specific CD4^+^ T cells. This is important because recent work shows that CD40-CD40L signaling does more than provide costimulation: it can program long-lasting functional changes in human monocytes, similar to trained immunity (40). We found that CD40L^+^ polyfunctional CD4^+^ T cells are the main responding population after both IIV4 and RZV, and that these responses persist with aging, suggesting that CD40-CD40L interactions may be a common control point linking innate and adaptive immunity after vaccination. RZV, in particular, induces high levels of CD40L^+^IL-2^+^TNF^+^IFN-γ^+^CD4^+^ T cells in both age groups. In contrast, weaker CD4^+^ T cell responses to IIV4 may reflect limited CD40-driven support, helping explain its lower cellular immunogenicity and reduced efficacy in older individuals.

Intriguingly, both IIV4 and RZV modulated the circulating inflammatory proteins, particularly in older individuals. We observed different effects of the IIV4 vaccines administered in fall 2021 and fall 2022 on the proteins in the circulation of older adults, arguing for variation due to differences in the antigenic composition of the vaccines. However, the absence of a placebo group for the 2022/2023 season and the relatively small sample size limit the conclusions. Notably, RZV reduced several inflammatory proteins in older adults, with stronger effects after the second dose. This age-dependent response may be due to the inherently lower baseline levels of inflammatory mediators in young adults.

Consistent with previous studies, we found higher concentrations of inflammatory markers in older adults than in young adults. This is likely partly due to the higher rates of comorbidities and risk factors, such as hypertension and cardiovascular diseases (e.g., atherosclerosis) (41, 42). Elevated concentrations of inflammatory mediators in older adults indicate a state of chronic low-grade inflammation, commonly referred to as “inflammaging” (43). This persistent inflammation may impair the immune system’s ability to mount a robust antigen-specific response upon vaccination, possibly due to immune exhaustion or overactivation (44) due to continuous stimulation leading to diminished function and reduced responsiveness to new antigens. These mediators might also trigger regulatory feedback that weakens immune responses. Lowering systemic inflammation could prevent immune exhaustion and improve both innate and adaptive responses. As an example, the Bacillus Calmette-Guérin vaccine reduced systemic inflammation in a Dutch cohort of healthy participants, which was associated with trained immunity induction — the capacity of innate immune cells to develop a memory-like response (45). Consistently, our study found a strong negative association between inflammatory protein concentrations in the blood and antibody fold-increases in both vaccine groups.

In our study cohort, baseline inflammation was generally negatively correlated with PBMC-derived IFN-γ response after RZV, while it showed a positive association with IFN-γ response after IIV4. Interestingly, in the older volunteers vaccinated with IIV4, higher circulating concentrations of CXCL9, CXCL10, and IFN-γ were linked to higher IFN-γ production. In the old RZV group, higher CXCL9 and CXC10 were also correlated with greater antibody response. Since CXCL9 and CXCL10 are key chemokines involved in activating NK cells and Th1 immunity, it is tempting to speculate that they contribute to the enhanced type II IFN response observed after vaccination (46) and possibly to improved antibody production. Because this study only identifies associations but does not establish causation, future research should aim to understand better how these proteins affect vaccine responses, especially in different age groups. Additionally, future studies should explore strategies to modulate inflammatory proteins prior to or during vaccination by improving or developing adjuvants, with the aim of enhancing vaccine efficacy, especially in older adults.

This study demonstrated the moderate influence of IIV4 and RZV on immune cell subsets. IIV4 significantly increased WBCs in young adults, driven by lymphocyte and monocyte numbers and sustained until D180, whereas older adults showed elevated lymphocytes only at D7. On the other hand, RZV caused transient but stronger changes in cell counts in both age groups. A temporary increase in neutrophil and monocyte numbers and infiltration to the injected muscle has been reported after other vaccinations, suggesting immediate innate immune system activation and response (47, 48). The similar magnitude of changes in immune cell counts between young and older adults suggests that the AS01-adjuvanted RZV can achieve comparable initial innate immune cell activation in both age groups, supporting its effectiveness (34).

It is important to note that the RZV and RZV-associated placebo injections were administered during spring/summer (first and second dose), whereas IIV4 and IIV4-associated placebo injections were administered during fall. Seasonal variations are known to influence immune function and therefore vaccine-induced responses. Although the study design partially accounted for this by including 2 young placebo groups vaccinated in either spring or fall, helping to control for background seasonal effects, we cannot fully determine the impact of seasons on vaccine responses.

A limitation of this study is that a placebo group was only available for young participants, whereas older adults were all vaccinated due to ethical considerations. As a result, direct comparisons between vaccinated and unvaccinated older individuals could not be made, and age-related differences in immune responses must therefore be interpreted with some caution. Particularly for IIV4, where vaccine responses are strongly influenced by prior influenza infections and vaccination history, including an unvaccinated control group of older adults would have helped to better distinguish vaccine effects from those related to preexisting immunity.

In summary, this study highlighted the age-dependent vaccine responses after IIV4 and RZV exposure, focusing on cellular and humoral immunity, inflammatory mediators, and the influence of systemic inflammation prior to vaccination. Although RZV induced potent humoral and cellular immune responses while decreasing systemic inflammation in older adults, IIV4 was able to induce strong antibody production with weaker cellular responses. The incapacity of IIV4 to induce specific cellular immune responses may contribute to its year-dependent suboptimal efficacy. Our findings emphasize the role of adjuvants such as AS01 in enhancing vaccine responses in older adults, with modulating low-grade inflammation being a potential strategy. Further research is needed to explore the underlying transcriptional and epigenetic mechanisms driving different vaccine responses by RZV compared with IIV4. Understanding the immunological pathways contributing to increased vaccine immunogenicity could help refine existing adjuvants and develop new ones.

## Methods

### Sex as a biological variable.

Both men and women were included in the clinical study. Sex was considered as a confounder to investigate the differences in vaccine responses in young and older individuals.

### Clinical study.

The volunteer recruitment in this single-center, randomized, partially placebo-controlled, open-label study was conducted at and sponsored by the Radboud University Medical Center between September 2021 and May 2023. Volunteers aged 18–35 and those 60 years or older were eligible to participate. Exclusion criteria were the use of systemic immunomodulatory drugs, acute or active illness within 2 weeks before the study, receipt of any vaccination within 4 weeks before or after the start of the study, receipt of a herpes zoster vaccination in the past year, known allergy to the components of IIV4 or RZV, being immunocompromised, and pregnancy or breastfeeding. Young female participants had to have a negative pregnancy test before participating.

After signing the informed consent form, young participants were randomized to receive either IIV4, RZV, IIV4-associated placebo, or RZV-associated placebo (3:3:1:1) (Figure 1A). Older participants were randomized to receive either IIV4 or RZV (1:1); no placebo was administered to older individuals for ethical considerations because these vaccines are recommended to this population. Blood samples were collected at baseline, and volunteers were vaccinated with either a placebo (0.9% NaCl solution), IIV4 (Fluarix Tetra, GSK, UK, 2021/2022 and 2022/2023 seasons), or RZV (Shingrix, GSK). IIV4 vaccinations took place between September and January during the influenza season, whereas RZV doses were administered between April and July 2022. Corresponding placebo groups were included in the same period. All vaccinations were administered in the morning between 8:00 am and 12:00 pm as a 0.5 mL intramuscular injection into the deltoid muscle. The RZV and associated placebo groups received a second dose 2 months after the first injection. Study participants were followed for 6 months after the first visit in the IIV4 and associated placebo group and 8 months after the first visit in the RZV and associated placebo group (6 months after the second injection). The blood collection times and readouts for each time point are shown in Figure 1B. All adverse events and potential immune-mediated disorders were recorded throughout the study. Solicited adverse events recorded in patient diaries within 7 days after vaccinations and SAEs throughout the clinical study are reported in this manuscript.

Of note, all young and 10 of the 27 older participants in the IIV4 group were vaccinated with IIV4 season 2021/2022, and the remaining 17 older participants were injected with the IIV4 season 2022/2023 vaccine. These two vaccines had 2 common viral strains, A/Victoria/2570/2019 IVR-215 H1N1 (referred to as A/Victoria/H1N1 herein) and the B/Phuket/3073/2013 WT (B/Yamagata lineage) (referred to as B/Phuket herein). Additionally, the 2021/2022 season contained the A/Tasmania/503/2020 IVR-221 H3N2 (referred to as A/Tasmania/H3N2 herein) and B/Washington/02/2019 WT (B/Victoria lineage) (referred to as B/Washington herein); the 2022/2023 season included A/Darwin/6/2021 IVR-227 H3N2 (referred to as A/Darwin/H3N2in herein) and B/Austria/1359417/2021 BVR-26 (B/Victoria lineage) (referred to as B/Austria herein).

### Sample collection.

Blood samples were collected by venipuncture into EDTA and serum tubes. To obtain plasma and serum from the EDTA and serum tubes, respectively, blood was centrifuged for 10 minutes at 2,700*g* at room temperature. Serum was used for HAI and anti-gE antibody measurements; plasma was used to measure protein concentrations. All samples were stored at –80°C until analysis.

### Measurement of immune cell counts.

The number of WBCs, monocytes, neutrophils, and lymphocytes was determined from whole blood using a hematology analyzer (Sysmex). This analyzer operates on principles similar to those of a flow cytometer: it uses forward-scatter light to determine cell volume, side-scatter light to identify cell nuclei and granules, and side fluorescence to detect nucleic acids and organelles.

### HAI measurements.

HAI antibody titers were determined on sera before and 60 days after vaccination using the method derived from the WHO Manual on Animal Influenza Diagnosis and Surveillance (49). Measurements were conducted on thawed frozen serum samples with a standardized and validated methodology. Briefly, serum samples were treated with receptor-destroying enzyme (Sigma-Aldrich, C8772-1VL) overnight to remove nonspecific serum inhibitors, diluted to 1:10, and serial diluted 2-fold in duplicate from 1:10 to 1:10,240. After adding an equal volume of standardized virus (4 HAU/25 μL), neutralization was performed for 1 hour at room temperature, followed by the addition of the RBCs. Of note, HAU stands for hemagglutinating unit, which is the highest dilution of the virus causing complete hemagglutination. After 60–120 minutes, plates were tilted, and the HAI titer was defined as the reciprocal of the last serum dilution that fully inhibits hemagglutination as compared with an RBC control well. Each sera sample was tested in duplicate within the same assay. The titer results were reported as the geometric mean titer of the duplicates. The assay positivity cutoff value was 10 (1/dilution) and was confirmed for each strain.

### Anti-VZV gE antibody measurements.

Serum anti-gE antibody concentrations were measured from sera before vaccination and 60 days after each RZV dose using a validated GSK in-house ELISA, with a technical cutoff of 97 mIU/mL, as previously described (39). Of note, mIU, milli-international unit, is a standardized quantity of a biological activity or effect. Briefly, GSK-produced, purified recombinant VZV gE was precoated on a polystyrene 96-well microplate at a 2 μg/mL final concentration. The wells were washed and blocked with BSA. Diluted serum samples were added and incubated for 1 hour at room temperature. In the next step, the plates were washed again and goat antibodies against human IgG conjugated to HRP (a-IgG-HRP conjugate, Kirkegaard and Perry Laboratories, catalog 214-1002) were added. After incubation for an hour at room temperature and subsequent washing, a chromogen-substrate solution (3,3’,5,5’-tetramethylbenzidine and hydrogen peroxide) was added. The reaction was stopped with sulfuric acid, and the optical density was measured at 450 nm against the reference wavelength, which was 620 nm, using an Emax microplate reader (Molecular Devices). The assay was calibrated against the VZV WHO international reference.

### PBMC isolation.

Venous blood collected with EDTA tubes was diluted with PBS, and PBMCs were isolated using density gradient centrifugation with Ficoll-Paque (GE HealthCare) in SepMate tubes (Stemcell Technologies). After centrifugation at 1,200*g* for 10 minutes, the upper layer containing PBMCs was collected and washed twice with cold PBS. The cells were suspended in RPMI 1640 medium (Dutch modification) (Thermo Fisher Scientific) supplemented with 1 mM sodium pyruvate (Thermo Fisher Scientific), 2 mM GlutaMAX (Thermo Fisher Scientific), and 50 μg/mL gentamicin (Centrafarm), and then counted using the hematology analyzer. Of note, the Dutch-modified RPMI medium has HEPES and a lower concentration of sodium bicarbonate compared with a classical RPMI medium (1 g/L instead of 2 g/L). PBMCs were frozen at 20 × 10^6^ cells/mL in Recovery Cell Culture Freezing Medium (Thermo Fisher Scientific) and stored at –150°C until further use.

### Ex vivo stimulation of cytokine production.

Frozen PBMCs were thawed in a warm RPMI medium containing 10% calf serum (Capricorn Scientific) and supplemented as described in *PBMC isolation*. After washing the cells twice to remove the residual freezing medium, cells were resuspended in RPMI medium with 10% pooled human serum. The pooled human serum was prepared in-house at the Radboud University Medical Center, using the serum provided by the Department of Medical Microbiology. Each serum sample was tested on human PBMCs, and the ones inducing or reducing an immune response were excluded. The remaining samples were pooled to be used for experiments.

Cells were counted and seeded into 96-well U-bottom plates (Sarstedt) at a density of 5 × 10^5^ cells/well. PBMCs were stimulated with 1 μg/mL of Fluarix Tetra (IIV4, 2021/2022 and 2022/2023 season) and 4 μg/mL of the gE component of Shingrix (RZV) or left unstimulated. After a 7-day incubation at 37°C, cell-free supernatants were collected.

IFN-γ concentrations in supernatants were measured using IFN-γ DuoSet ELISA kits (R&D Systems), following the manufacturer’s instructions. The same batch was used to perform ELISA for all samples, and the different time points for each donor were measured on the same plate. Moreover, 3 control samples with known cytokine concentrations were utilized in measurements to ensure that the reference standards worked correctly. The lower limit of detection for this assay was 93.76 pg/mL.

### Antigen-specific CD4^+^ T cell responses.

Antigen-specific T cell responses were measured by intracellular cytokine staining from PBMCs (50, 51). In short, thawed PBMCs (10^6^ cells/well) were stimulated in 96-well plates either with the A/H1N1/Victoria strain (1 μg/mL), the B/Phuket strain (1 μg/mL), pools of 15-mer peptides overlapping by 11 amino acids and covering the zoster gE sequence (1.25 μg/mL), or left unstimulated (medium only) as a negative control. After 2 hours of incubation at 37°C, GolgiPlug brefeldin A solution at 1/1,000 final dilution (BD Biosciences) was added overnight to inhibit cytokine secretion. Then, the cells were harvested, stained for surface markers (a viability dye, CD4 and CD8), and fixed and permeabilized with Cytofix/Cytoperm kit (BD Biosciences) per the manufacturer’s instructions. Intracellular staining was performed using the following markers: CD40L, IL-2, TNF, IFN-γ, IL-13, IL-17, and 4-1BB. After washing with perm/wash buffer (BD Biosciences), cells were analyzed by flow cytometry. Live cells that were positive for CD4^+^ 4-1BB^+^ and positive for at least 1 of the 4 markers (CD40L, IL-2, TNF, IFN-γ) were quantified. Since the expression of IL-13 and IL-17 was very low, these markers were not used for the analysis. The antigen-specific CD4^+^ T cell frequency was calculated as the difference between the frequency of CD4^+^ T cells stimulated with the antigen and those stimulated with medium alone. The frequency was expressed as the number of cells per 10^6^ CD4^+^ T cells. Details on the antibodies used in flow cytometry staining are in Supplemental Table 8.

### Proximity extension assay measurements of circulating inflammatory mediators.

Circulating proteins in the plasma were measured by proximity extension assay using the Olink Target 96 Inflammation Panel, which includes 92 proteins. All time points from the same participant were measured on the same plate to eliminate potential interplate variations in measurements and fold change calculations. Proteins were normalized based on the interplate controls, and expression levels were presented on a log_2_ scale called normalized protein expression.

The analysis excluded 18 of 92 proteins due to a more than 20% missing data frequency. After this exclusion, principal component analysis was performed, and 2 apparent outliers were removed. The R package *limma* (v3.60.4) was then utilized to conduct differential expression analysis between the age groups and across different time points within the same group.

### Trademarks.

Shingrix and Fluarix Tetra are trademarks owned by or licensed to GSK. AS01 is a GSK proprietary adjuvant system.

### Statistics.

This clinical trial was designed as an exploratory study; hence, no formal sample size calculations were made. Data comparing young and older participants were adjusted for confounding variables of sex and BMI. Comparisons between age groups were made using the Mann-Whitney *U* test; comparisons within the same group across different time points utilized Wilcoxon’s signed rank test. When more than 2 time points were compared, Friedman’s test was applied. The baseline concentration of circulating proteins was correlated with fold changes in antibody concentrations and IFN-γ responses using Spearman’s correlation. The statistical methods applied to each graph are detailed in the figure legends. A *P* value of less than 0.05 was considered statistically significant.

For statistical tests and data visualization, GraphPad Prism (v10) and RStudio (Posit PBC, v4.4.1) were used. Dot plots were created with Prism (v10), and heatmaps and volcano plots were generated in RStudio using the *pheatmap* (v1.0.12), *ggplot2* (v3.5.1), and *ggrepel* (v0.9.5) packages.

### Study approval.

The local ethics committee, Medical Research Ethics Committee (MREC) Oost-Nederland, granted ethical approval for this study (NL76061.091.20). Written informed consent was received from all participants prior to inclusion. All experiments were conducted under the Declaration of Helsinki. The trial is registered at ClinicalTrials.gov (NCT05082688).

### Data availability.

All the data that support the findings of this study are available from the corresponding author upon reasonable request. The numerical data underlying the figures are reported in the Supporting Data Values file.

## Author contributions

GK, EJMT, WB, SMB, MMM, and MGN conceptualized the study and designed the experiments. GK, EJMT, and EAD were responsible for the overall conduct of the clinical study, administration, and approvals. GK, EJMT, LSH, and EAD were involved in the collection and processing of biological samples. GK, LSH, BG, LVE, HL, and SB performed the experiments and curated the data. GK and AB performed data analysis and visualization. GK, MO, YN, WB, SMB, MMM, and MGN were involved in data interpretation. JTO, MMM, and MGN supervised the study. GK prepared the original draft. All authors read, edited, and accepted the final version.

## Conflict of interest

MMM is a scientific founder of Lemba Therapeutics. MGN is a scientific founder of Lemba Therapeutics, BioTrip, Trained Therapeutix Discovery, and Salvina Therapeutics. SB, AB, WB, and SMB are employed by and hold financial equities in GSK.

## Funding support

- GSK (to MMM and MGN).
- European Research Council, ERC Advanced grant 833247 (to MGN).
- Netherlands Organization for Scientific Research (NWO), Spinoza grant (to MGN).

## Supplementary Material

Supplemental data

Supporting data values

## Figures and Tables

**Table 1:**
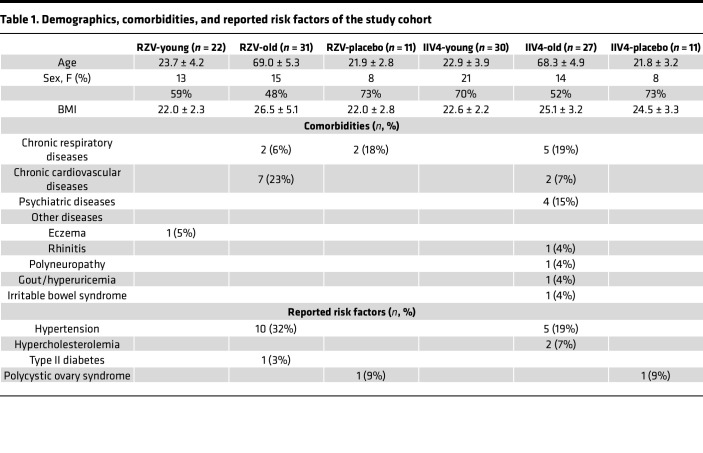
Demographics, comorbidities, and reported risk factors of the study cohort

**Table 2:**
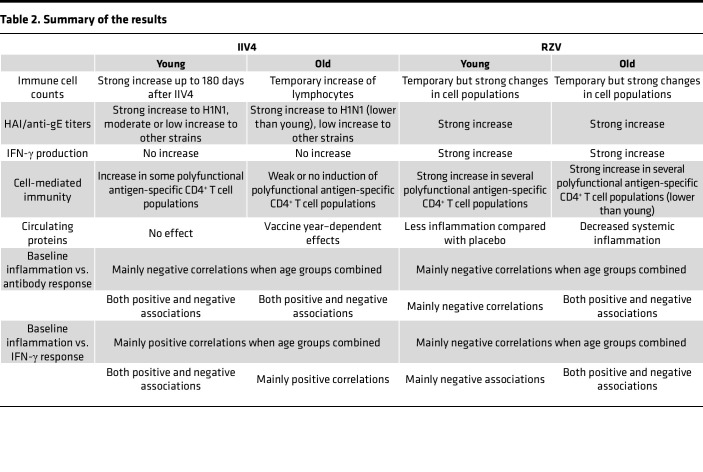
Summary of the results

## Version 1 06/02/2026

In-Press Preview

## Version 2 07/22/2026

Electronic publication
