# Supplementary material for: Age-related differences in immune responses to inactivated influenza and adjuvanted recombinant herpes zoster vaccines
Source: JCI Insight. 2026 Jun 2;11(14):e195618. doi: 10.1172/jci.insight.195618 (PMC13461205; doi:10.1172/jci.insight.195618)
Supplement: Supplementary file 1 [file jciinsight-11-195618-s031.pdf]

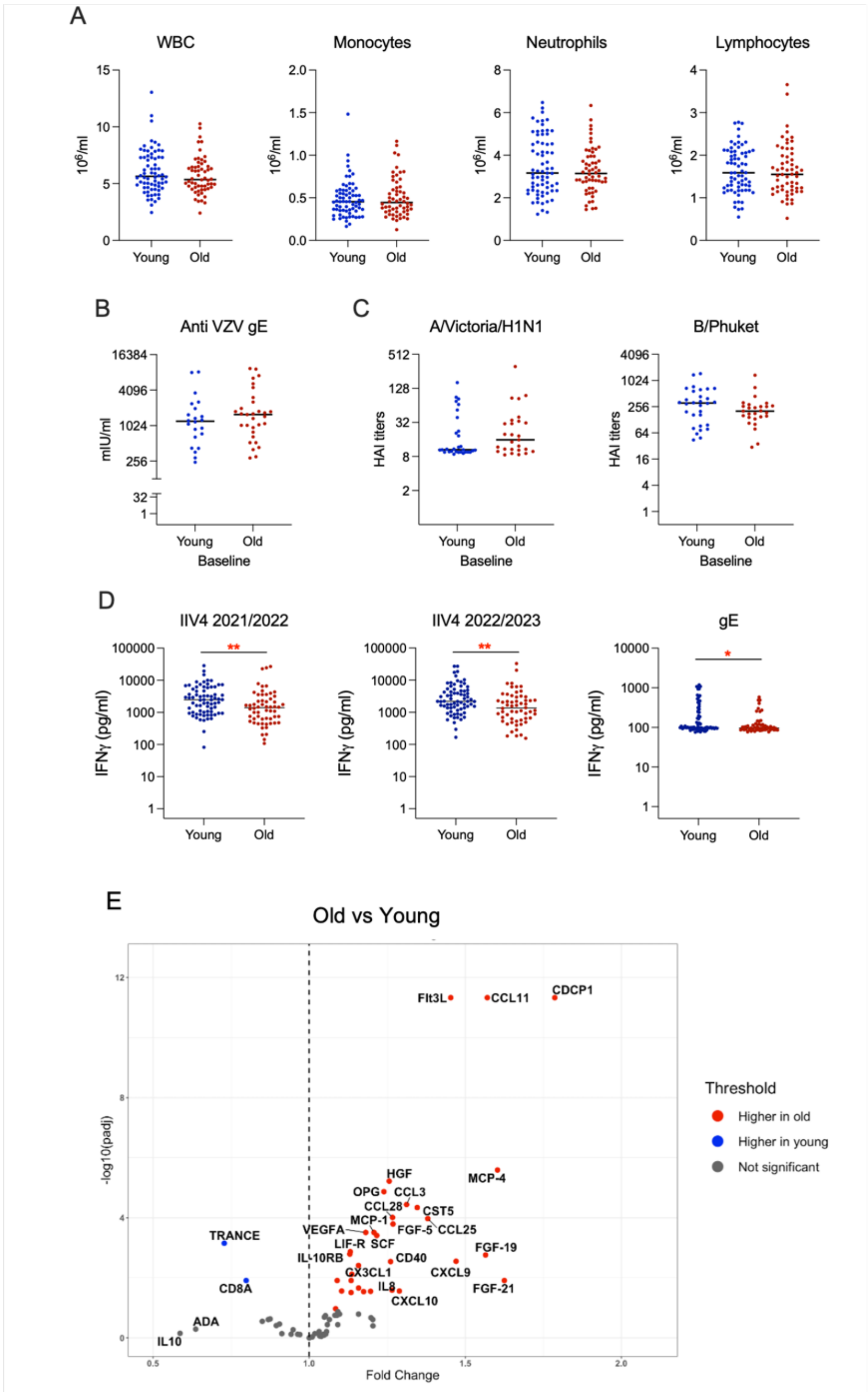

Supplementary Figure 1: Baseline immune parameters of the young and older individuals in the study cohort.

A) White blood cell (WBC), neutrophil, monocyte and lymphocyte counts. B) Anti-VZV gE antibody levels. C) Hemagglutination inhibitory antibody titers against A/Victoria/H1N1 and B/Phuket strains. The y-axis

values in panels B and C are displayed on a log2 scale. D) IFNγ production of PBMCs after a 7-day incubation with 1 μg/ml of IIV4 season 2021/2022, 1 μg/ml of IIV4 season 2022/2023 and 4 μg/ml of the gE antigen from RZV. The y-axis values in panel D are displayed on a log10 scale. The LLOD of the assay is 93.76 pg/ml. E) Circulating inflammatory proteins measured by Olink were compared between young (18-35 years) and older (≥60) adults. Age groups were compared using the Mann-Whitney test. The p-values in panel E were adjusted using the Benjamini-Hochberg method. LLOD: Lower limit of detection. *$p<0.05$, **$p<0.01$.

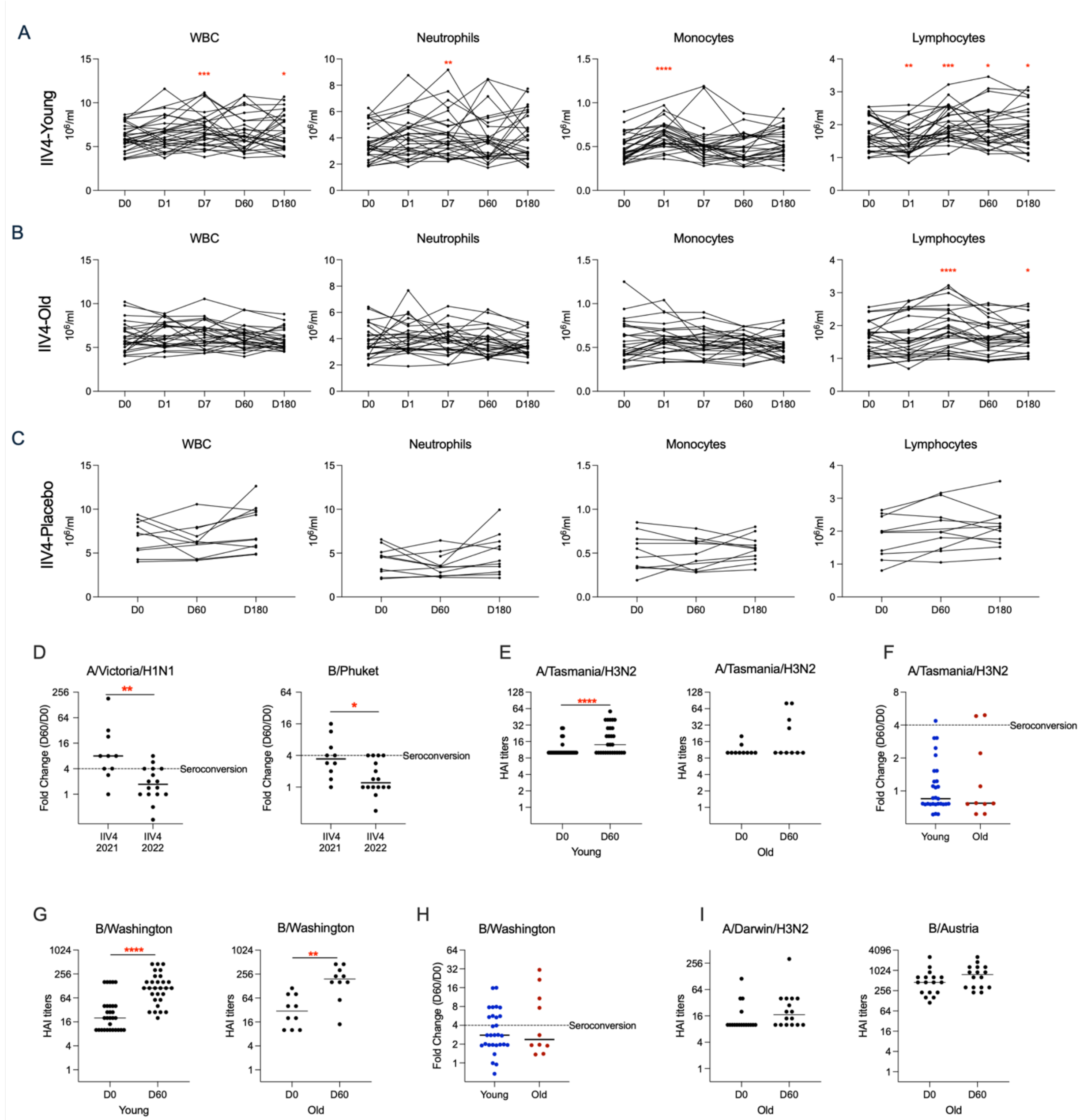

Supplementary Figure 2: Absolute immune cell counts and adaptive immune responses after IIV4 vaccination in young and older adults.

Changes in WBC, neutrophil, monocyte and lymphocyte counts after IIV4 vaccination in A) young and B) older adults and C) placebo injection in the placebo group. D) Comparison of fold HAI titers in older adults vaccinated with IIV4 2021 and IIV4 2022 to the A/Victoria/H1N1 and B/Phuket. E) HAI titers and F) comparison of fold changes (D60/D0) in antibody production against the A/Tasmania/H3N2 strain. G) HAI titers and H) Comparison of fold changes (D60/D0) in antibody production against the B/Washington strain. I) HAI titers against the A/Darwin/H3N2 and B/Austria strains before and 60 days after IIV4 exposure in older individuals. In panels E to H, only the antibody data of older individuals who received

the IIV4 2021/2022 season were shown, as those strains are only present in this season's vaccine. Panel I only shows the data of older adults who received the IIV4 2022/2023 season since all young participants were vaccinated with the IIV4 2021/2022 season. The y-axis values in panels D-I are displayed on a log2 scale. In panels A-C, the post-vaccination timepoints were compared with before vaccination (D0) using the Wilcoxon signed-rank test. In other panels, different time points within the same group were compared using the Wilcoxon signed-rank test, while fold changes between young and older adults were compared using the Mann-Whitney test. *p<0.05, **p<0.01, ***p<0.001, ****p<0.0001.

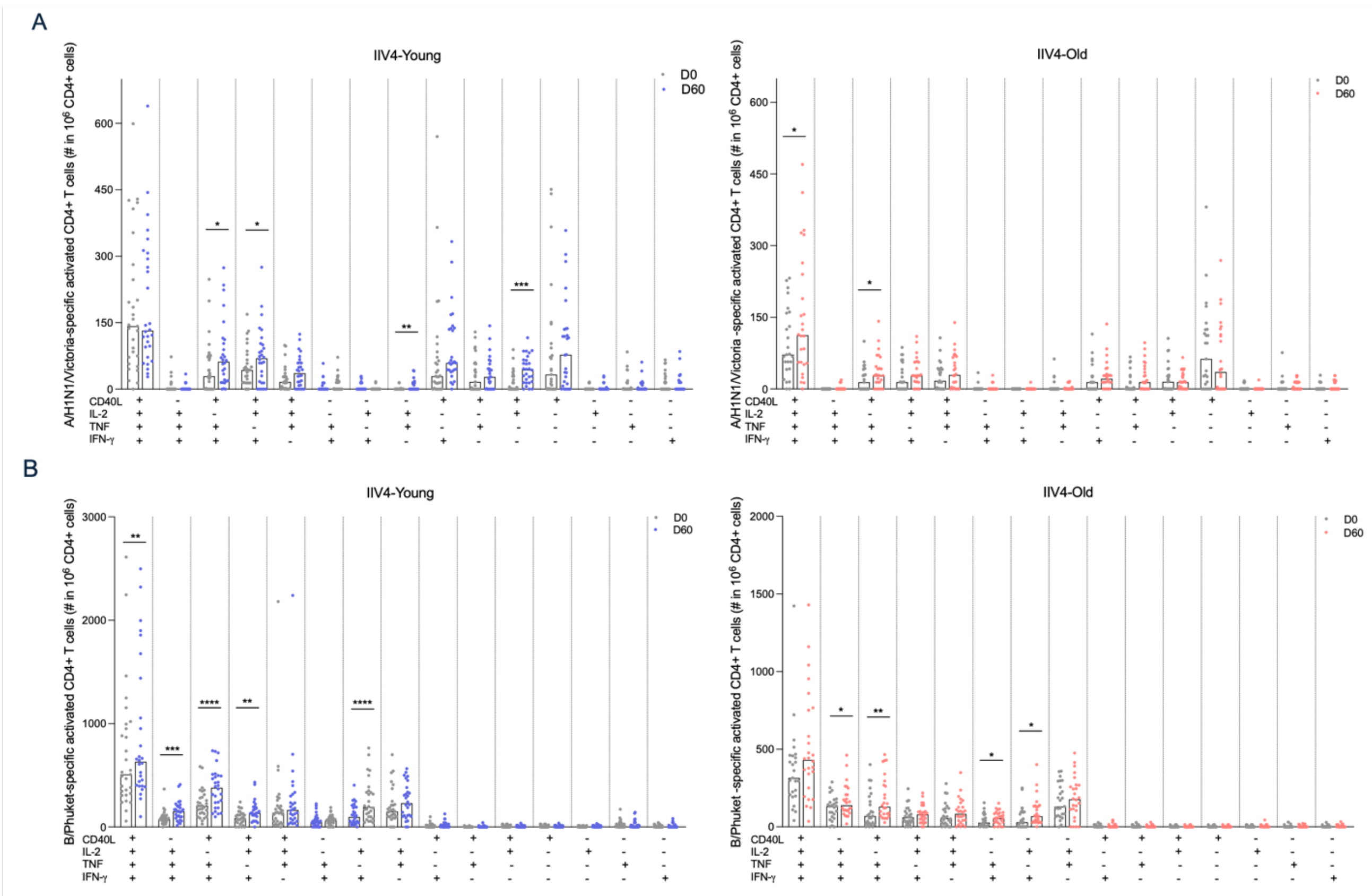

Supplementary Figure 3: Antigen-specific CD4+ T cell response after IIV4 in young and older adults.

The number of activated CD4+ T cells per 10^6 CD4+ T cells that are positive for CD40L, IL-2, TNF, and IFN-γ after incubation with A) A/H1N1/Victoria strain and B) B/Phuket strain. For each combination of 4 markers, D0 was compared to D60 using the Wilcoxon signed-rank test. *p<0.05, **p<0.01, ***p<0.001. ****p<0.0001.

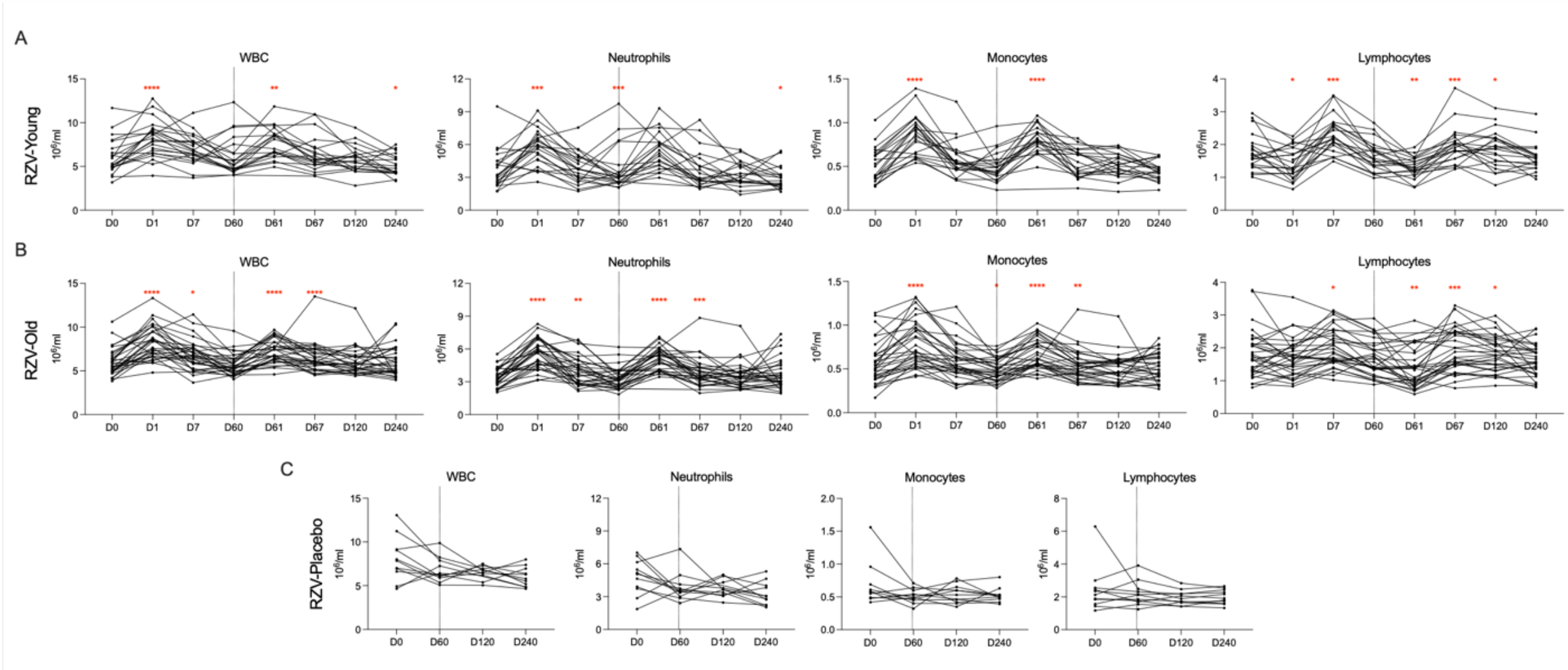

Supplementary Figure 4: Absolute immune cell counts after RZV and placebo vaccination in young and older adults.

Changes in WBC, neutrophil, monocyte and lymphocyte counts after RZV vaccination in A) young and B) older adults and C) placebo injection. The participants were vaccinated with a placebo or RZV at both D0 and D60. Timepoints up to and until the 2nd RZV dose (D1, D7 and D60) were compared to D0, while D61, D67, D120 and D240 were compared to D60 (2nd RZV dose) using the Wilcoxon signed-rank test. *p<0.05, **p<0.01, ***p<0.001, ****p<0.0001.

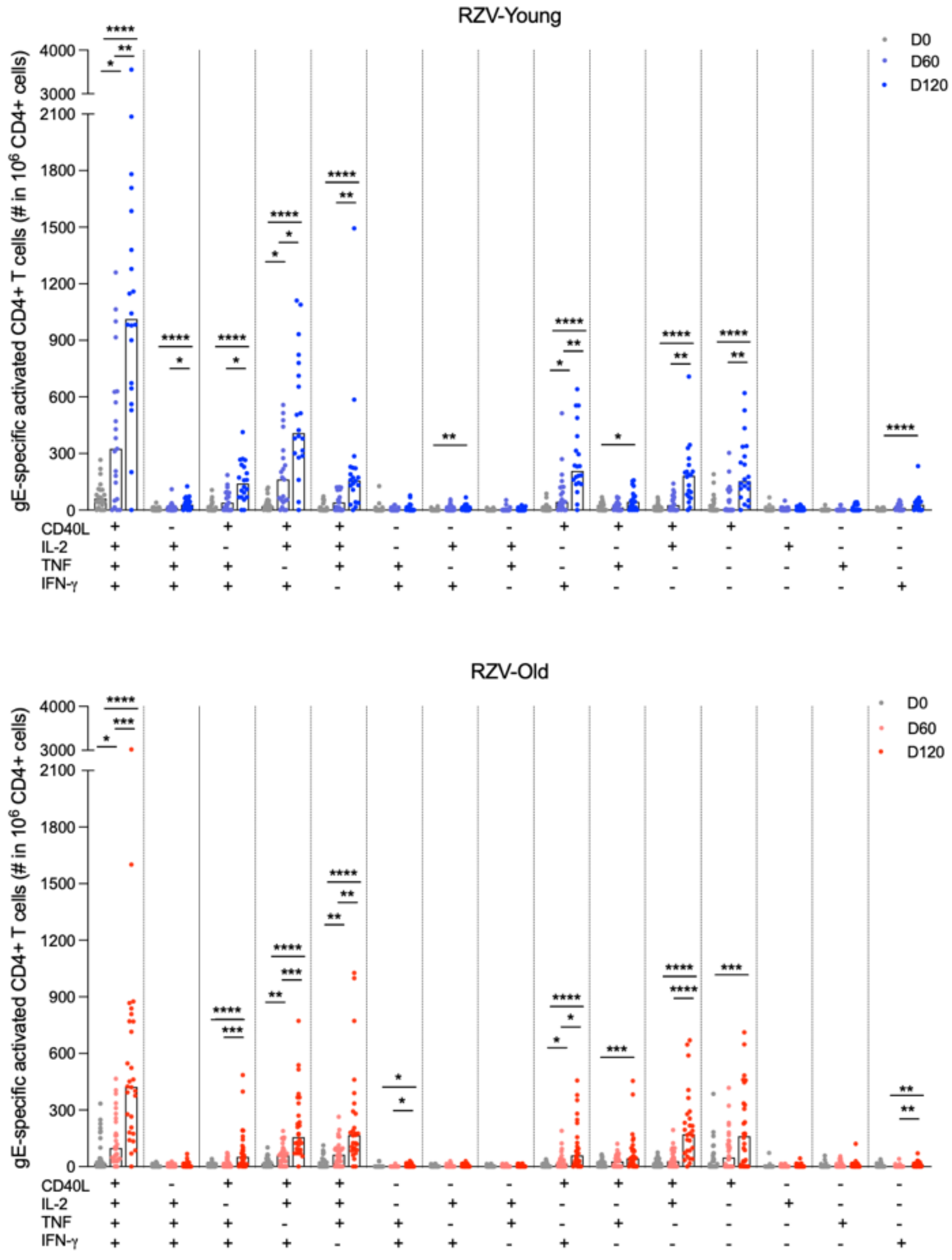

Supplementary Figure 5: Antigen-specific CD4+ T cell response after RZV in young and older adults.

The number of activated CD4+ T cells per 10^6 CD4+ T cells that are positive for CD40L, IL-2, TNF, and IFN-γ after incubation with the gE-antigen. For each combination of 4 markers, the timepoints were compared to each other using Dunn's multiple comparisons test. *p<0.05, **p<0.01, ***p<0.001. ****p<0.0001.

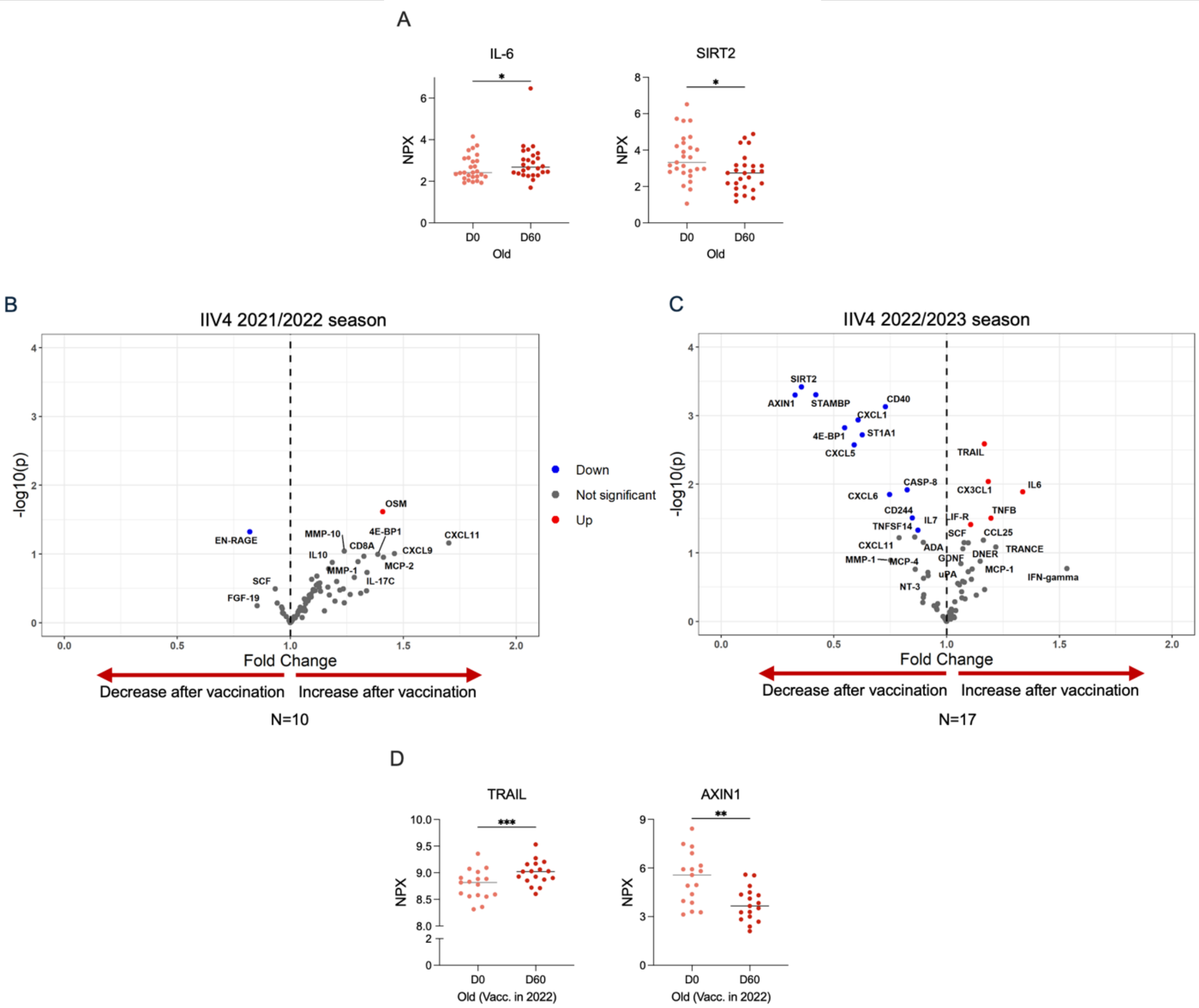

Supplementary Figure 6: Year-dependent effect of IIV4 in the proteome of older adults.

A) Example dot plots of IL-6 and SIRT2 concentrations in older adults before and 60 days after IIV4 exposure. Volcanos displaying the changes in proteins in the older adults who received the B) IIV4 2021/2022 season (n=10) and C) IIV4 2022/2023 season (n=17) Blue dots represent the proteins significantly downregulated after vaccination, while red dots show the significantly upregulated proteins (nominal p-value<0.05). D) Example dot plots of TRAIL and AXIN1 concentrations in older adults who received the IIV4 2022/2023 season before and after vaccination. NPX: Normalized protein expression. In panels A and D, comparisons were made using the Wilcoxon signed-rank test. *p<0.05, **p<0.01.

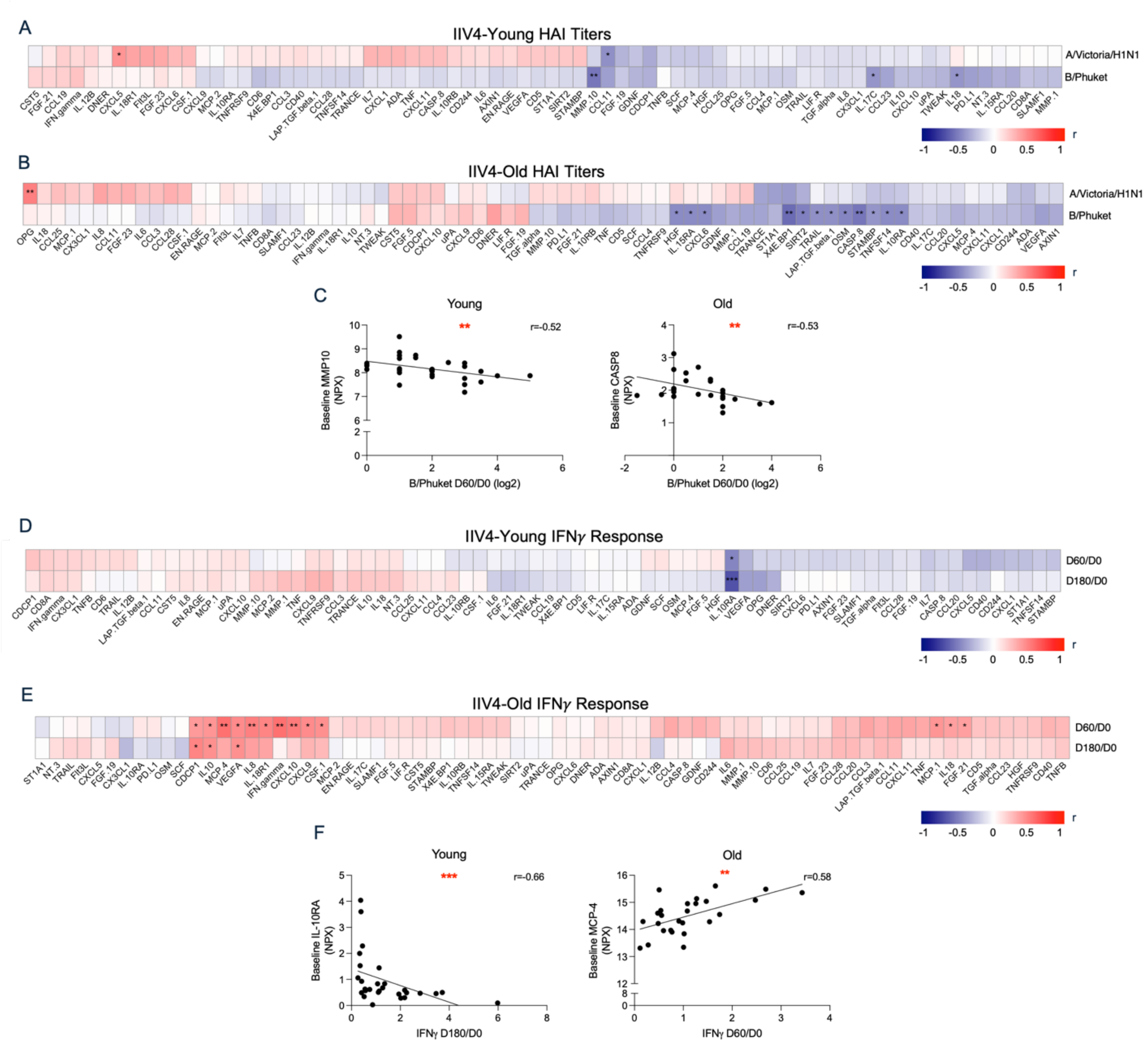

Supplementary Figure 7: Correlation between circulating proteins at baseline and IIV4-induced immune responses in the young and old age groups.

Heatmaps depicting the association between baseline concentrations of circulating immune-related proteins and the fold antibody response (D60/D0) against the A/Victoria/H1N1 and B/Phuket strains following IIV4 vaccination in A) young and B) older adults. C) Example correlation plots showing the relationship between baseline MMP10 with the fold antibody response to B/Phuket in young adults and CASP8 with the fold antibody induction to B/Phuket in older adults. Heatmaps illustrating the correlation between baseline circulating immune-related proteins and the fold IFNγ response (D60/D0 and D180/D0) from PBMCs after a 7-day stimulation with 1 µg/ml of IIV4 in D) young and E) older adults. F) Example correlation plots of baseline IL-10RA levels with D180/D0 fold IFNγ induction in the young group and MCP4 with D60/D0 fold IFNγ production. The scale bars in panels A, B, D, and E, along with the ”r” values on the correlation graphs, represent the correlation coefficients. Spearman’s correlation analysis

was performed for all correlation graphs shown in this figure. Stars on heatmaps indicate the nominal p-values. NPX: Normalized protein expression. *$p<0.05$, **$p<0.01$, ***$p<0.001$.

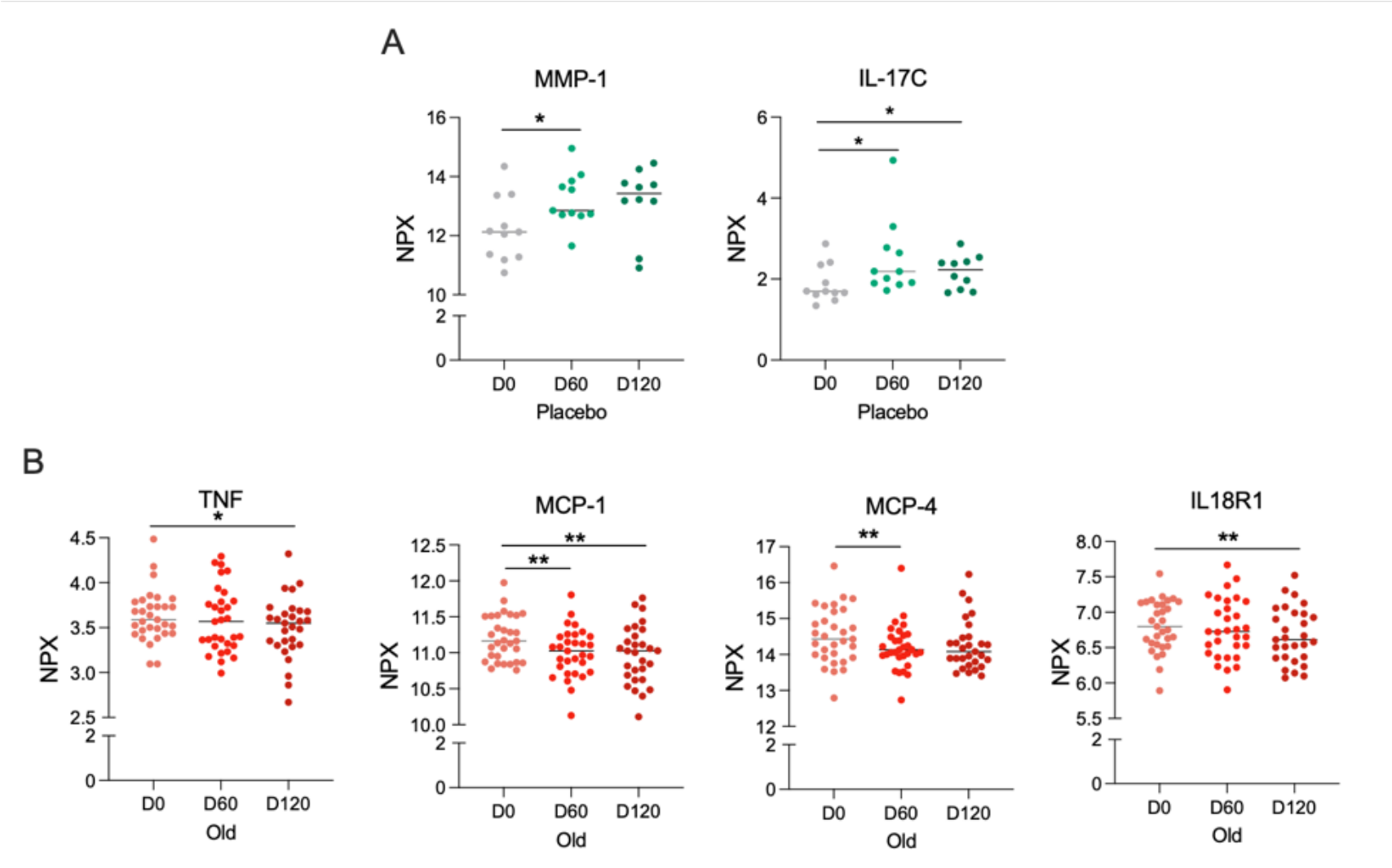

Supplementary Figure 8: Example dot plots of proteins after vaccination in the placebo and RZV-Old groups.

A) MMP-1 and IL17-C concentrations in the RZV-Placebo group before and after vaccination. B) TNF, MCP-1, MCP-4 and IL18R1 concentrations in older adults before and after RZV exposure. NPX: Normalized protein expression. D60 and D120 timepoints were compared to before vaccination (D0) using the Wilcoxon signed-rank test. *p<0.05, **p<0.01.

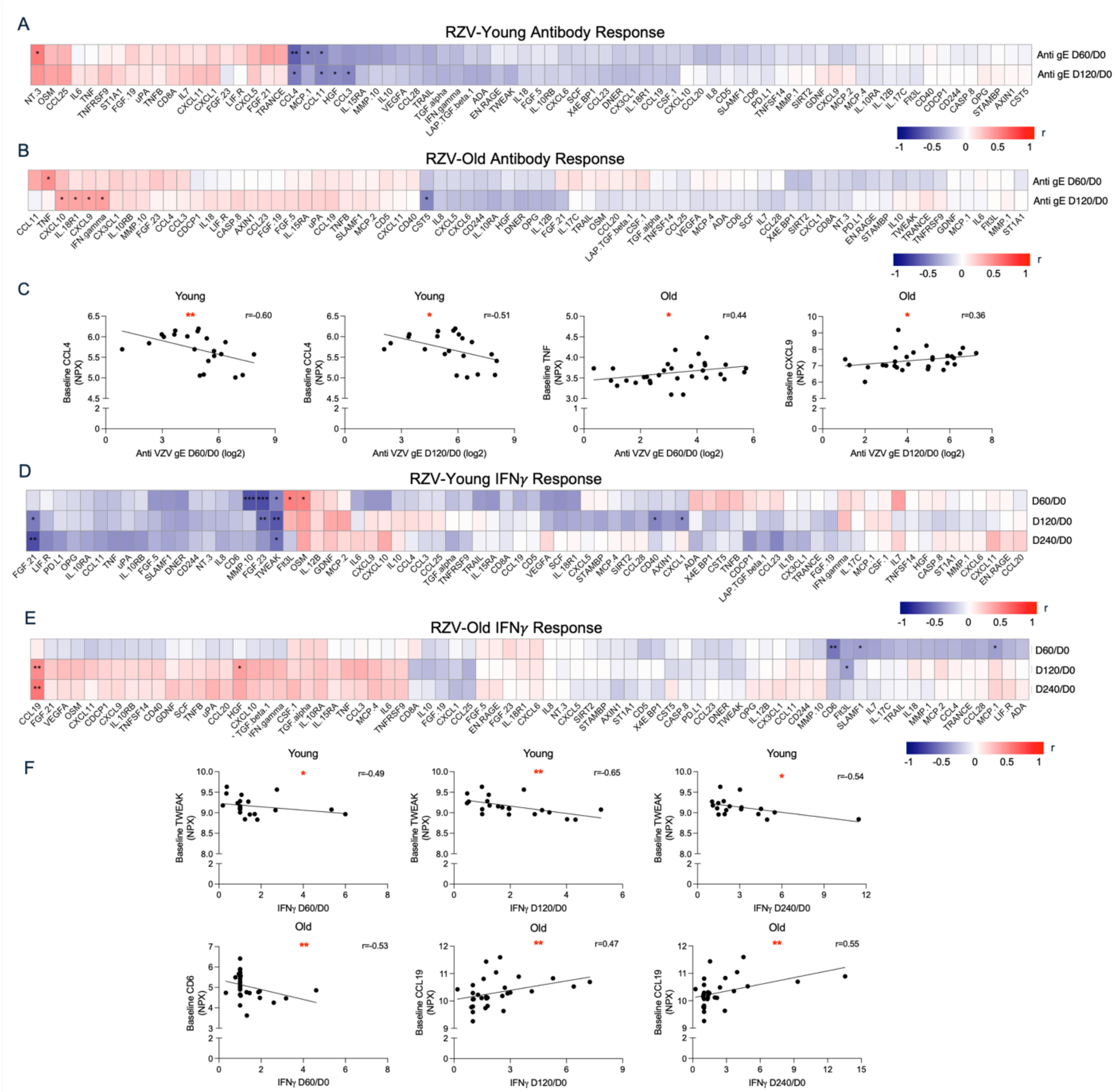

Supplementary Figure 9: Correlation between circulating proteins at baseline and RZV-induced immune responses in the young and old age groups.

Heatmaps depicting the association between baseline concentrations of circulating immune-related proteins and the fold antibody response (D60/D0 and D120/D0) following RZV vaccination in A) young and B) older adults. C) Example correlation plots showing the relationship between baseline levels of CCL4 with the fold antibody response after the first (D60/D0) and second dose (D120/D0) in young adults and the association between baseline TNF and CXCL9 concentrations with the D60/D0 and D120/D0 antibody response, respectively, in older adults. Heatmaps illustrating the correlation between baseline circulating immune-related proteins and the fold IFNγ response (D60/D0, D120/D0 and D240/D0) from PBMCs after a 7-day stimulation with 4 µg/ml of the gE antigen in RZV in D) young and E) older adults. F) Example correlation plots of baseline TWEAK levels with fold IFNγ response in young

adults and CD6 with D60/D0 and CCL19 with D120/D0 and D240/D0 fold IFNγ production in older adults. The scale bars in panels A, B, D, and E, along with the ”r” values on the correlation graphs, represent the correlation coefficients. Spearman’s correlation analysis was performed for all correlation graphs shown in this figure. Stars on heatmaps indicate the nominal p-values. NPX: Normalized protein expression. *p<0.05, **p<0.01, ***p<0.001.

Supplementary Tables:

Supplementary Table 1: Adverse reactions occurred within 7 days after the first RZV or a placebo dose.

RZV 1st Dose	RZV-Young n/total	RZV-Young %	RZV-Old n/total	RZV-Old %	Placebo n/total	Placebo %
**Any reaction**	22/22	100%	29/31	94%	6/11	55%
**Injection site reactions**	22/22	100%	29/31	94%	3/11	27%
**Pain**	22/22	100%	29/31	94%	2/11	18%
**Redness**	4/22	18%	7/31	23%	1/11	9%
**Swelling**	4/22	18%	7/31	23%	0/11	-
**Systemic reactions**	21/22	95%	18/31	58%	5/11	45%
**Fatigue**	15/22	68%	12/31	39%	3/11	27%
**Myalgia**	15/22	68%	10/31	32%	2/11	18%
**Joint pain**	2/22	9%	5/31	16%	0/11	-
**Headache**	10/22	45%	9/31	29%	2/11	18%
**Fever**	5/22	23%	2/31	6%	0/11	-
**Gastrointestinal symptoms**	4/22	18%	3/31	10%	0/11	-
**Malaise**	10/22	45%	9/31	29%	1/11	9%
**Cold shivers**	6/22	27%	7/31	23%	1/11	9%

Supplementary Table 2: Adverse reactions occurred within 7 days after the second dose of RZV or a placebo.

RZV 2nd Dose	RZV-Young		RZV-Old		Placebo	
	n/total	%	n/total	%	n/total	%
**Any reaction**	21/21	100%	26/31	84%	4/10	40%
**Injection site reactions**	21/21	100%	26/31	84%	2/10	20%
**Pain**	19/21	90%	25/31	81%	2/10	20%
**Redness**	8/21	38%	8/31	26%	0/10	-
**Swelling**	7/21	33%	4/31	13%	0/10	-
**Systemic reactions**	19/21	90%	18/31	58%	3/10	30%
**Fatigue**	15/21	71%	11/31	35%	2/10	20%
**Myalgia**	18/21	86%	8/31	26%	2/10	20%
**Joint pain**	2/21	5%	5/31	16%	0/10	-
**Headache**	16/21	76%	7/31	23%	2/10	20%
**Fever**	10/21	48%	6/31	19%	0/10	-
**Gastrointestinal symptoms**	6/21	29%	5/31	16%	1/10	10%
**Malaise**	13/21	62%	9/31	29%	0/10	-
**Cold shivers**	11/21	52%	3/31	10%	0/10	-

Supplementary Table 3: Adverse reactions occurred within 7 days after the IIV4 vaccination.

IIV4	IIV4-Young		IIV4-Old		Placebo	
	n/total	%	n/total	%	n/total	%
**Any reaction**	27/30	90%	19/27	70%	8/11	73%
**Injection site reactions**	24/30	80%	14/27	52%	4/11	36%
**Pain**	24/30	80%	14/27	52%	4/11	36%
**Redness**	3/30	10%	2/27	7%	1/11	9%
**Swelling**	2/30	7%	2/27	7%	0/11	-
**Systemic reactions**	21/30	70%	13/27	48%	7/11	64%
**Fatigue**	12/30	40%	6/27	22%	4/11	36%
**Myalgia**	15/30	50%	7/27	26%	3/11	27%
**Joint pain**	2/30	7%	0/27	-	0/11	-
**Headache**	10/30	33%	4/27	15%	1/11	9%
**Fever**	0/30	-	2/27	7%	0/11	-
**Gastrointestinal symptoms**	3/30	10%	3/27	11%	1/11	9%
**Malaise**	6/30	20%	6/27	22%	2/11	18%
**Cold shivers**	4/30	13%	3/27	11%	1/11	9%

Supplementary Table 4: Grade 3 adverse reactions occurred within 7 days after the first RZV vaccination.

RZV 1st Dose -Grade 3	RZV-Young		RZV-Old		Placebo	
	n/total	%	n/total	%	n/total	%
**Any reaction**	8/22	36%	3/31	10%	0/11	-
**Injection site reactions**	6/22	27%	0/31	-	0/11	-
**Pain**	4/22	18%	0/31	-	0/11	-
**Redness**	2/22	9%	0/31	-	0/11	-
**Swelling**	1/22	5%	0/31	-	0/11	-
**Systemic reactions**	7/22	32%	3/31	10%	0/11	-
**Fatigue**	3/22	14%	2/31	6%	0/11	-
**Myalgia**	4/22	18%	0/31	-	0/11	-
**Joint pain**	0/22	-	1/31	3%	0/11	-
**Headache**	0/22	-	1/31	3%	0/11	-
**Fever**	1/22	5%	0/31	-	0/11	-
**Gastrointestinal symptoms**	0/22	-	0/31	-	0/11	-
**Malaise**	1/22	5%	0/31	-	0/11	-
**Cold shivers**	1/22	5%	0/31	-	0/11	-

Supplementary Table 5: Grade 3 adverse reactions occurred within 7 days after the second RZV vaccination.

RZV 2nd Dose -Grade 3	RZV-Young		RZV-Old		Placebo	
	n/total	%	n/total	%	n/total	%
**Any reaction**	6/21	29%	5/31	16%	0/10	-
**Injection site reactions**	2/21	10%	3/31	10%	0/10	-
**Pain**	2/21	10%	2/31	6%	0/10	-
**Redness**	1/21	5%	1/31	3%	0/10	-
**Swelling**	0/21	-	0/31	-	0/10	-
**Systemic reactions**	6/21	29%	3/31	6%	0/10	-
**Fatigue**	3/21	14%	1/31	3%	0/10	-
**Myalgia**	3/21	14%	0/31	-	0/10	-
**Joint pain**	0/21	-	0/31	-	0/10	-
**Headache**	1/21	5%	0/31	-	0/10	-
**Fever**	2/21	10%	1/31	3%	0/10	-
**Gastrointestinal symptoms**	1/21	5%	0/31	-	0/10	-
**Malaise**	2/21	10%	1/31	3%	0/10	-
**Cold shivers**	1/21	5%	0/31	-	0/10	-

Supplementary Table 6: Grade 3 adverse reactions occurred within 7 days after the IIV4 vaccination.

IIV4 – Grade 3	IIV4-Young		IIV4-Old		Placebo	
	n/total	%	n/total	%	n/total	%
**Any reaction**	2/30	7%	1/27	4%	1/11	9%
**Injection site reactions**	0/30	-	0/27	-	0/11	-
**Pain**	0/30	-	0/27	-	0/11	-
**Redness**	0/30	-	0/27	-	0/11	-
**Swelling**	0/30	-	0/27	-	0/11	-
**Systemic reactions**	2/30	7%	1/27	4%	1/11	9%
**Fatigue**	2/30	7%	1/27	4%	0/11	-
**Myalgia**	0/30	-	0/27	-	0/11	-
**Joint pain**	0/30	-	0/27	-	0/11	-
**Headache**	0/30	-	0/27	-	0/11	-
**Fever**	0/30	-	0/27	-	0/11	-
**Gastrointestinal symptoms**	0/30	-	0/27	-	1/11	9%
**Malaise**	0/30	-	1/27	4%	1/11	9%
**Cold shivers**	0/30	-	1/27	4%	0/11	-

Supplementary Table 7: Serious adverse events recorded throughout the study.

Serious Adverse Events (SAEs)	RZV-Young	RZV-Old	RZV-Placebo	IIV4-Young	IIV4-Old	IIV4-Placebo
**#SAEs**	1/22	2/31	0/11	0/30	0/27	0/11
**Road traffic accident**	1/22	-	-	-	-	-
**Acute heart failure**	-	1/31	-	-	-	-
**Atrial fibrillation**	-	1/31	-	-	-	
**SAEs associated with vaccination**	-	-	-	-	-	-
**Deaths**	-	1/31	-	-	-	-

Supplementary Table 8: Antibodies used in the flow cytometry staining.

**Immune marker/antibody**	**Fluorophore**	**Provider**	**Clone**
CD3	BV605	BioLegend	OKT3
CD4	BB700	BD	SK3
CD8	APC-H7	BD	SK1
IL-2	APC-R700	BD	MQ1-17H12
TNFa	PeCy7	BD	Mab11
IFNy	FITC	BD	4S.B3
CD40L (CD154)	PE	Miltenyi Biotech	REA238
IL-13	APC	BioLegend	JES10-5A2
IL-17	BV785	BioLegend	BL168
4-1BB (CD137)	BV421	BioLegend	4B4-1
Live&Dead	Aqua	Thermo Fisher	
